# Human Papillomavirus and Other Relevant Issues in Cervical Cancer Pathogenesis

**DOI:** 10.3390/ijms26125549

**Published:** 2025-06-10

**Authors:** Amitabha Ray

**Affiliations:** School of Health Professions, D’Youville University, 320 Porter Ave, Buffalo, NY 14201, USA; rayam@dyc.edu; Tel.: +1-(716)-829-8243

**Keywords:** cervical lesions, HPV, social factors, tobacco, hormones, cervicovaginal bacteria

## Abstract

Cancer of the uterine cervix (cervical cancer) is a leading cancer among women worldwide, although its incidence has been reducing in many developing nations. In the majority of cervical cancer cases, the presence of high-risk human papillomavirus (HPV) is usually detected. However, a growing body of evidence currently considers that exclusive HPV infection may not be sufficient for cancer development. Apart from certain common risk factors for cervical cancer, like poor nutritional status and smoking, many studies documented an association with other viral infections, such as human immunodeficiency virus (HIV) and herpes simplex virus type 2 (HSV-2). Similarly, vaginal bacterial populations perhaps play a key role in cervical cancer. It may be worth mentioning that different bacterial species can immensely influence (either protecting or adversely) the biochemical characteristics of the cervicovaginal environment—for example, *Lactobacillus crispatus*, *Gardnerella vaginalis*, and *Chlamydia trachomatis*. As a result, chronic infections with unfavorable microorganisms (other than HPV) may affect the pathological processes of malignancy. On the other hand, the cervix is an estrogen-sensitive organ like the corpus uteri (i.e., the body of the uterus). Estrogen and different estrogen receptors are implicated in the development and promotion of various cancers, including endometrial cancer. A number of reports also suggest a close association between estrogen and HPV in the development of cervical cancer. Furthermore, estrogen is linked with the characteristics of the vaginal microenvironment including bacteria. Therefore, several of the abovementioned factors (some are preventable) could play an important role in the progression of cervical neoplastic lesions.

## 1. Introduction

Cancer of the uterine cervix (or cervical cancer) is the fourth most common cancer among women worldwide, but there is a wide geographical variation in incidence rates. Recently, we noticed a decreasing trend in this cancer burden, while higher incidence rates have been recorded in countries such as Eswatini (Swaziland), Lesotho, and Zimbabwe [1,2]. In cervical cancer, women of lower socioeconomic status are usually affected, and human papillomavirus (HPV) infection possibly plays a major role in the etiology of this disease [3]. As per the Centers for Disease Control and Prevention (CDC), approximately 91% of cervical cancer cases are associated with any HPV type. It may be worth mentioning that at least 14 types (i.e., high-risk types) out of more than 200 types of HPV are believed to cause malignant transformation. However, two special types (HPV-16 and -18) are frequently detected in cervical precancerous lesions (dysplasia/cervical intraepithelial neoplasia or CIN) as well as in full-blown cancer. According to the World Health Organization (WHO), these two HPV types are responsible for 70% of cervical cancer and precancerous lesions. It is well known that HPV can spread by skin/mucosal contact, especially through sexual transmission, and HPV is one of the most common sexually transmitted viral diseases throughout the world. It is also believed that in the majority of people with HPV infection, the immune system can naturally clear the virus [4,5]. As a result, only a few women with persistent high-risk HPV infection eventually develop cervical cancer. On the other hand, a large number of HPV types that belong to the low-risk group may cause benign warts.

Overall, papillomaviruses, including human pathogenic types, are relatively small non-enveloped viruses with an icosahedral capsid of around 55 nm diameter, and the viral genome consists of circular double-stranded DNA of approximately 8000 base pairs (bp). We can divide the HPV genome into three main portions: an early (E) region that encodes nonstructural proteins, a late (L) region that encodes capsid proteins, and a noncoding long control region (LCR) that regulates viral replication and gene expression [6]. In the beginning, actively dividing basal epithelial cells are infected by HPV; subsequently, the virus particles advance to the surface along with the differentiated cells, wherein they replicate. Finally, viral late genes are expressed, as well as progeny viruses are generated and released into the environment (HPVs are nonlytic viruses) [7]. Nevertheless, as indicated earlier, the viral genome encodes six early proteins (E1, E2, E4, E5, E6, and E7), which play roles in virus replication and cell transformation, and two late (L1 and L2) proteins form the capsid [8]. The E6 and E7 proteins are believed to be responsible for the malignant transformation of cervical epithelial cells: E6 and E7 inactivate p53 and the retinoblastoma protein (pRB), respectively [9]. Of note, both p53 and pRB are important tumor suppressor proteins.

## 2. Risk Factors

**Figure 1:**
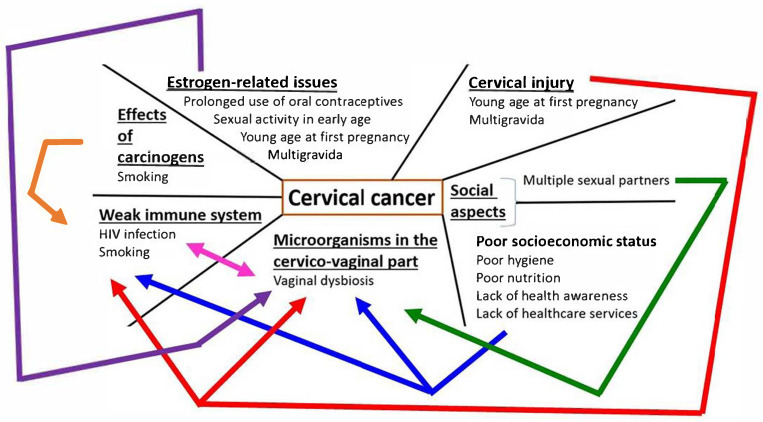
A general overview of different risk factors for cervical cancer.

Different scientific organizations, such as the American Society of Clinical Oncology, American Cancer Society, and CDC, as well as several authors [10,11], have tried to compile various risk factors for cervical cancer comprehensively. We may categorize these risk factors into four primary groups: (I) A weak immune system that might be caused by various pathological agents and circumstances, for example, HIV infection (acquired immunodeficiency syndrome/AIDS caused by the human immunodeficiency virus/HIV), poor nutritional status (could be due to economic condition/poverty), and smoking, which can weaken the immune system by various tobacco-associated toxic chemicals such as nicotine, free radicals, and polycyclic aromatic hydrocarbons. It is well known that tobacco-associated chemicals are also linked to certain pathologic phenomena like oxidative stress and DNA damage, as well as numerous diseases [12,13]. (II) Presence of other microorganisms in the cervical area, such as genital herpes (HSV), chlamydia infection, and vaginal bacterial population. (III) Estrogen-related issues, e.g., prolonged use of oral contraceptives (birth control pills), sexual activity at an early age (particularly less than 18 years), young age at first pregnancy and multigravida, which can also cause cervical injury. (IV) Social aspects, for instance, include having many sexual partners and poor socioeconomic status, such as the inability to maintain proper hygiene, lack of health awareness, and inaccessibility of suitable healthcare services. Obviously, certain risk factors fall into more than one category/group. For example, poor nutritional status generally relates to both social aspects and weak immunity, and promiscuity may invite undesirable microorganisms into the cervicovaginal region (Figure 1).

## 3. Screening Methods and Some Related Issues

The screening of cervical precancerous conditions constitutes an important aspect of secondary prevention. However, several issues are associated with it. Compared to high-income countries, the burden of cervical cancer is at least 10-fold higher in low- and middle-income countries [14]. Moreover, according to WHO, nearly 90% of deaths occur in low- and middle-income nations. Precise diagnostic tests for all sexually transmitted diseases are extensively employed in high-income nations. These tests are particularly advantageous for the detection of asymptomatic cases, such as chronic infection with high-risk HPV types and early stages of the pre-neoplastic condition. Unfortunately, in low- and middle-income countries, these diagnostic tests are mostly unavailable, apart from the difficulties in therapeutic management and follow-up. Therefore, in these countries, the screening methods are usually indirect.

**Figure 2:**
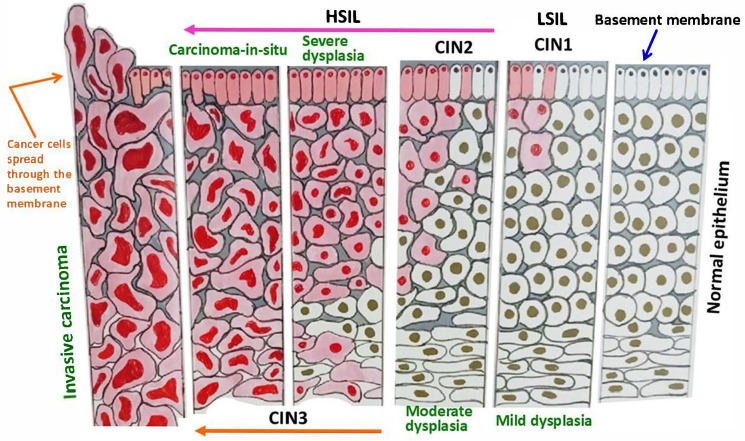
Progression of the cervical lesion to invasive carcinoma. CIN: Cervical intraepithelial neoplasia, HSIL: High-grade squamous intraepithelial lesion, LSIL: Low-grade squamous intraepithelial lesion.

It is worth mentioning that the carcinogenic process commonly starts from the area of the squamocolumnar junction (or transformation zone) of the cervix uteri. Nonetheless, the pathological processes generally take several years from dysplasia to invasive cancer (Figure 2). Therefore, older women are usually at the highest risk, and clearly, regular screening is beneficial to prevent disease progression. On the other hand, in the United States, the most common HPV-related neoplastic disease is oropharyngeal cancer (squamous cell type), which consists of nearly 25% of all head and neck malignancies, and a similar trend is also noticed in other regions of North America and Northern Europe [15,16,17]. Regarding the epidemiological behavior and risk factors, there are differences between cervical cancer and HPV-related oropharyngeal cancer. The majority of this oropharyngeal cancer originates at the deep level of tonsillar crypts (specifically from the lingual and palatine tonsillar areas among Caucasian men younger than 50 years) and displays a basaloid character, association with HPV-16, and overexpression of p16(INK4a), a cell cycle regulator and tumor suppressor [18,19,20,21]. Hence, the detection of the p16 protein by immunohistochemistry is a trustable marker in this disease, unlike the Pap smear or related tests for cervical lesions. Of note, an increased cellular level of HPV E7 competes with the transcription factor E2F for binding with the pRB, and thus hinders the normal functional balance among E2F, pRB, and the cyclin-dependent kinase inhibitor p16, which finally accumulates in HPV-infected cells.

In regions where appropriate screening methods such as colposcopy and cytopathology are not available, visual inspection of the uterine cervix with acetic acid (VIA) or Lugol’s iodine (VILI or Schiller’s test) has been suggested. The VIA test is performed by application of freshly prepared 4% acetic acid in the cervical area, and assessment is performed with a bright light source (like a halogen lamp) after 1 min. The test is considered positive when a dense acetowhite area (with distinct and regular margins) linked with the squamocolumnar junction appears [22]. The precise mechanism of the acetowhite appearance is not clear. However, in dysplastic/neoplastic cells after acetic acid application, the color change could be due to increased wide-angle side scattering of light from both the nucleus (more scattering) and the cytoplasm, in contrast to normal cells [23]. On the other hand, the application of 5% Lugol’s iodine solution on the cervix induces color changes depending on glycogen, which is present in the squamous epithelium (brown/black appearance) while glycogen is not present in columnar cells (unstained), and precancerous or cancer cells have little or no glycogen (saffron/yellow color) [24].

Ideally, any prevention programs for cervical cancer in both developing and developed countries should be based on the Pap test. In this test, cells were collected from the squamocolumnar junction/transformation zone by slowly rotating a spatula 360° one time (or by cytobrush). The collected material is thinly and evenly spread on a glass slide and fixed immediately with 95% ethanol or a spray fixative (polyethylene glycol with alcohol). The staining technique for the Pap test was developed by George Papanicolaou in 1942; however, the procedure has undergone several modifications. The basic principle of staining is to distinctly differentiate the cell populations of basophilic and acidophilic groups along with their precise chromatin pattern [25].

Detection methods such as the HPV DNA test, co-testing (DNA test along with a Pap smear), the polymerase chain reaction (PCR) test, or hybrid capture technology are clearly beyond the financial capability of most of the world’s population. Available tests, such as visual inspection and cytology screening, are linked with several issues, e.g., inadequate sensitivity, variability of the assay system, including manpower quality, and opportunity for repeated testing. Clearly, ineffective screening cannot prevent disease progression satisfactorily. Studies have identified a group of high-risk HPV-positive women who displayed normal cervical cytology [26,27,28]. Among this group, there is a higher possibility for poor adherence to screening protocols, a decrease in attendance, and low re-testing, which can lead to an increased risk of cervical lesions.

## 4. HPV-Negative Cervical Cancer

In several cervical cancer cases, investigators were unable to detect HPV. The number of these HPV-negative cases varies widely in different studies. For example, Lee et al. reported that 3–8% of cervical cancer lesions are HPV-negative [29]. On the other hand, Tjalma documented that 15% of cervical cancers were HPV-negative in Belgium [30]. In a study in Hong Kong, the investigators did not find HPV DNA in 21.5% of cases [31]. Although several propositions have been put forward for the negative findings, such as HPV genome loss and technical problems, the variations could be due to differences in geographical regions and ethnic groups [29,32]. Of note, the majority of the data were obtained from Caucasian women. Furthermore, it must be considered that the presence of HPV DNA in a specimen by the PCR technique cannot make a distinction between a transient infection and viral involvement in tumorigenesis [29,33]. Reasonably, improved identification methods or better nucleic acid amplification can detect more viral particles. A steady reduction in the number of HPV-negative lesions was observed in a meta-analysis conducted on data published from 1990 to 2010, which included 243 studies and 30,848 invasive cervical cancer patients [34]. On the other hand, the findings from a cohort of 965,360 women aged 30–64 (Kaiser Permanente Northern California database) suggested that even when the accompanying HPV test was negative, the risks for CIN grade 3 (CIN3) or cervical cancer (CIN3+) were higher following a Pap result of atypical glandular cells or atypical squamous cells, cannot exclude high-grade squamous intraepithelial lesion/HSIL (ASC-H) [35]. In a population-based screening study (POBASCAM) in the Netherlands, the investigators selected 18,448 women, and follow-up samples were collected for 14 years [36]. A higher CIN3+ risk was noticed in HPV-negative women who had a positive HPV test or a positive co-test in the previous round.

In general, elderly women are at higher risk of developing HPV-negative cervical cancer, and histologically, HPV-negative adenocarcinoma occurs commonly compared to squamous cell carcinoma [37]. It is believed that glandular epithelium (of adenocarcinoma) does not favor an effective HPV infection in comparison to squamous epithelium [30]. Nevertheless, a study from Spain analyzed 214 cervical cancer specimens, and 21 cases (10%) were HPV-negative [38]. Interestingly, among these HPV-negative malignancies, twelve cases had squamous cell carcinoma, six tumors were diagnosed as adenocarcinoma, two tumors were neuroendocrine carcinoma, and the remaining one lesion was adenosquamous carcinoma. Of note, the most common histological group is squamous cell cancer, which accounts for about 75–90% of invasive cervical cancer cases [30]. The second group is adenocarcinoma, which currently constitutes roughly 20% to 25% of cases. Intriguingly, this type represented only 5% during 1950–1960 [39]. The prevalence rate of the third group, i.e., adenosquamous carcinoma, ranges within 3–10% of all cervical cancers [40]. Histologically, adenosquamous carcinoma is a mixture of glandular and squamous components—so, carefulness is necessary to classify this variety. The remaining group is a collection of various rare cervical cancers that include lymphoma, sarcoma, and neuroendocrine tumors.

Neuroendocrine tumors of the uterine cervix are highly aggressive diseases, and they originate from the cells of embryonic neuroectoderm [41]. In general, neuroendocrine tumors represent approximately 1% to 1.5% of cervical cancer cases, and the primary histological subtype is small-cell neuroendocrine carcinoma [42]. The majority of small-cell neuroendocrine tumors of the cervix have been reported to be associated with HPV infections, usually with HPV-18 [43]. Regarding histopathological appearance, cervical small-cell neuroendocrine carcinoma resembles small-cell carcinoma of the lung [44]. Moreover, the commonly used chemotherapy regimen for these cervical tumors follows the therapeutic regimen for neuroendocrine tumors of the lung [45]. Both cervical and pulmonary neuroendocrine tumors are currently classified into four categories: carcinoid tumors, atypical carcinoid tumors, small-cell carcinoma, and large-cell carcinoma [46,47]. A brief account has been provided in Table 1 primarily on HPV-link to cervical small-cell tumors, along with the viral status of pulmonary small-cell carcinoma and neuroendocrine tumors of other sites that have no direct connection to the external environment (e.g., pheochromocytoma of the adrenal glands and pancreatic neuroendocrine tumors), unlike the gastrointestinal tract and lungs—the predominant sites for the development of such tumors [48,49,50,51,52,53,54,55,56,57,58,59,60,61,62,63,64,65,66,67,68,69,70,71,72,73,74,75,76]. Compared to cervical neuroendocrine tumors (~90% HPV positivity), Table 1 shows a considerably lower frequency of HPV detection in small-cell lung cancer. In addition, the presence of HPV in pheochromocytoma or pancreatic neuroendocrine tumors was not reported by any available studies.

Neuroendocrine cells can be detected in a normal endocervix, squamous cell carcinoma, and adenocarcinoma [42,77]. Similarly, neuroendocrine tumor cells can be present in premalignant lesions (CIN), squamous cell carcinoma, or adenocarcinoma [55,61,77]. Furthermore, distinguishing large-cell carcinoma of the cervix from poorly differentiated squamous carcinomas or adenocarcinomas may sometimes be difficult [77]. All these pathological characteristics could display the presence of HPV in neuroendocrine tumors that are admixed with HPV-associated adenocarcinoma and/or squamous cell carcinoma. On the other hand, practical significance should be considered when an advanced technique is used in either virus detection or identification of neuroendocrine molecules (for instance, by electron microscopy instead of immunohistochemistry) in order to show the increased rate of positivity. It may be worth mentioning that cancer cells often synthesize inappropriate or bizarre molecules owing to dysfunctional or imbalanced cellular machinery [78,79]. Nevertheless, the pathogenesis of neuroendocrine tumors is complicated and poorly understood. Interestingly, it is thought that irrespective of the anatomical site of origin, small-cell neuroendocrine tumors share similar histopathological features [53,80]. As already indicated above, there are similarities between cervical and pulmonary small-cell carcinomas. Of note, one of the most common neuroendocrine tumors is small-cell lung cancer; therefore, understanding its pathological processes is perhaps helpful in figuring out the molecular mechanisms of similar neoplastic growth. With respect to this, in a recent report, authors have described several risk factors for the development of neuroendocrine tumors, e.g., obesity, diabetes mellitus, smoking, and inflammatory conditions, apart from genetic factors [81]. Overall, the incidence of neuroendocrine tumors is on the rise throughout the world.

Like neuroendocrine tumors and contrary to the observation by Li and colleagues [34], it is also reported that in recent years, the incidence of HPV-negative cervical cancer has exhibited a rising trend [82]. Many scientists believe that the development of HPV-negative cancer follows a different tumorigenic pathway, as opposed to the pathogenesis of HPV-positive cancer [29,33,38,83]. In comparison to HPV-positive cases, a poor prognosis usually has been documented among patients with HPV-negative cervical cancer, and the pathological processes of HPV-negative malignancy have been recorded to be linked with a number of mutated genes, decreased DNA methylation, and disruptions in cell signaling that include WNT/β-catenin, Sonic Hedgehog, and phosphatidylinositol 3-kinase (PI3K) pathways [37,82,83]. In HPV-negative cancer, the mutated tumor suppressor p53 is one of the most frequently mentioned genes; a few others are PTEN, PIK3CA, KRAS, ARID1A, and CDKN2A. Furthermore, between HPV-positive and HPV-negative tumors, there are variations in the expression levels of different genes/proteins, for example, cell proliferation markers such as Ki67 and PCNA or growth factor receptors such as EGFR and HER2 [83]. Like the poorer disease outcomes of HPV-negative cervical cancer patients, as stated before, HPV-negative carcinomas of the other sites, such as the oropharynx, also show similar clinical characteristics compared to the relatively better prognostic condition of HPV-positive tumors [33,38].

It has been suggested that the combined effects of alcohol, tobacco, and HPV may be responsible for the pathogenesis of many oropharyngeal cancers [84]. In the same manner, the combination of different factors, e.g., a weakened immune system, long-term oral contraceptive use, multiparity, smoking, and concurrent infection with a sexually transmitted infectious agent and HPV, may promote the risk of cervical precancerous lesions [84]. On the other hand, Liu et al. commented that risk factors such as oldness, a weakened immune system, and sexually transmitted infection with *Chlamydia* could trigger the disease process of HPV-negative cervical cancer [82]. It is notable that in both HPV-positive and HPV-negative carcinogenic processes, a number of similar risk factors are involved.

## 5. Low-Risk HPV in Cervical Lesions

Investigators also documented the role of non-high/low-risk HPV in different cancers, including cervical precancerous lesions and cervical cancer (Table 2) [85]. In a study in Germany, out of a total of 5964 women, 293 women displayed infection with only a single type of HPV—either low-risk or undetermined-risk/novel types [86]. Among this group, there were 15 women with HSIL (carcinoma in situ: 2 cases), 8 with CIN3, and 7 with CIN2 lesions; 7 HSIL cases were associated with undetermined-risk HPVs and 8 with low-risk HPVs. Of note, HSIL comprises CIN2 and CIN3, moderate and severe dysplasia, and carcinoma in situ [87]. Nevertheless, in the aforementioned study, low-risk HPVs were detected in a considerable number of cases with abnormal cytological features, such as atypical squamous cells of undetermined significance and low-grade squamous intraepithelial lesions (LSIL) [86]. Similar findings were also noticed in a study of Slovenian women [88]. Furthermore, a number of studies observed the sole presence of low-risk HPV in both CIN/HSIL and cervical cancer specimens [89,90,91,92,93,94].

It is thought that 1–2% of primary cervical cancer cases are linked with low-risk HPV infection [37,94]. On the other hand, it is also believed that HPV-associated cancers only originate from infection with high-risk HPVs [84]. However, the pathological mechanisms related to low-risk HPV infection may be complex. Although there is a lesser risk of cancer development, low-risk HPV infection can cause the sequence of pathological changes from cytological abnormalities to HSIL and up to cancer [86,95,96]. It has been suggested that compared to high-risk HPV types, low-risk HPVs are less likely to integrate into the human genome, which is one of the important contributing factors to the development of cervical cancer [97,98]. Other risk factors, such as chemical carcinogens, alterations in the local microbiota, and immunological impairment, are likely to participate in low-risk HPV-linked tumorigenesis [98]. Interestingly, two low-risk HPV types—HPV-6 and HPV-11 were commonly isolated in various cancers, e.g., cervical cancer, laryngeal cancer, penile cancer, and anal cancer [92,95,97,99,100].

## 6. Influence of HIV and HSV-2

In a meta-analysis, the investigators examined the data from 236,127 women with AIDS in order to assess the association between HIV infection and cervical cancer in four continents (Africa, Asia, Europe, and North America) [101]. They observed that southern Africa and eastern Africa were the most affected geographical regions. HIV infection was detected in 63·8% of women with cervical cancer in southern Africa and 27·4% of women with cervical cancer in eastern Africa. It is worth mentioning that the prevalence of cervical cancer has been gradually increasing in sub-Saharan Africa, and HIV infection is an important accompanying factor. In developing countries, high-risk HPV infections are responsible for 7.7% of malignant diseases, particularly cervical cancer [102]. Another meta-analysis revealed that women with HIV infection had a higher risk of acquiring HPV, and this risk was inversely related to CD4 cell count [103]. On the other hand, a study was conducted during 2008–2012 in Lusaka, Zambia, and the study identified 224 HIV-positive cases out of 537 patients with stage I and II cervical cancer [104]. However, the study did not find any effects of HIV infection on cancer progression. In another study, in Rwanda, 42 women were diagnosed with CIN3 (i.e., 8.8%) out of 476 women who had both HPV and HIV infections [105]. Additionally, a study in Pune, India, showed that high-risk HPV types were present in 35.3% of women with HIV (*n* = 278) [106]. In addition, the study noticed that HPV-16 was the most prevalent type and was detected in 12% of all studied subjects. Usually, HPV-16 is the commonly isolated high-risk type in HPV-related neoplastic lesions.

In general, it has been estimated that roughly 77% of women with HIV are also HPV carriers [107]. So, globally, HIV-positive women have considerably elevated rates of HPV infections compared to women without HIV (Table 3) [108,109,110,111,112,113,114,115,116,117,118,119,120,121,122,123,124,125,126,127,128,129]. Unlike this type of clear association between HPV and HIV infection, the available reports on the role of herpes simplex virus (HSV) in HPV-related pathogenesis or cervical tumorigenesis are conflicting—some reports documented no or doubtful association [130,131,132,133]. Interestingly, HSV-2 infection significantly increases the risk of HIV acquisition [134]. Of note, the alpha subfamily of human herpes viruses includes HSV-1 (usually causes oral herpes), HSV-2 (usually causes genital herpes), and varicella-zoster virus—all these enveloped linear double-stranded DNA viruses live in our neurons, the site of their latent phase. In our body, these viruses can exist in either latent/dormant or lytic/active phases. According to WHO, approximately 3.7 billion individuals under the age of 50 (67% of the global population) had an HSV-1 infection in 2016. Among these people, about 192 million people aged 15–49 years had genital HSV-1 infections. Similarly, HSV-2 infections affected roughly 491.5 million (13.2%) people aged 15–49 years worldwide [135]. HSV infection can also cause severe complications such as encephalitis, eye infection, and neonatal infection from infected mothers.

However, in a report wherein the cross-sectional data of the National Health and Nutrition Examination Survey (NHANES) from 1999 to 2014 were analyzed, the authors observed that HSV-2 seropositivity was persistently associated with cervical cancer [136]. This study utilized a sample of 8184 female participants. Nonetheless, in a cross-sectional study on Mexican women who had cervical lesions with high-risk HPV, the investigators recorded that the risk of HSV-2 seropositivity was 1.7 times higher in HPV-positive cases, while HPV-positive cases were 9 times more susceptible to active HSV-2 infection in comparison with HPV-negative subjects [137]. In another study that evaluated blood and exfoliated cervical specimens of 1158 patients with invasive squamous cell cancer, 105 patients with adenocarcinoma and adenosquamous cancers, and 1117 controls, the investigators noticed in HPV-positive cases that HSV-2 seropositivity was associated with an increased risk of cancer development, particularly squamous cell cancer [138].

Interestingly, one Iranian study on cervical carcinoma tissue sections from 156 cases found that 71 cases (45.5%) were positive for HPV-16. Both HPV-16 and HSV-2 were positive only in 3 cases (2.3%); however, in these cases, the viral load of HSV-2 was very high compared to HPV-negative samples [139]. Furthermore, a study from China examined the cervical exfoliative cells for DNA of HPV-16, HPV-18, HSV-1, and HSV-2, from 24 squamous cell carcinoma patients, 210 CIN cases, 333 cervicitis, and 233 control women [140]. As compared to healthy controls, the presence of HPV was significantly higher in CIN and cancer, but not in cervicitis. Moreover, the prevalence of HSV-2, as well as coinfection by both HSV-2 and HPV, were higher in CIN and cancer cases than in controls. Therefore, the findings of these studies indicate a possible conjunction of pathologic effects of both HSV-2 and HPV in the development of cervical cancer. Nevertheless, selected reports from different investigators on the inconsistent role of HSV-2 in cervical cancer and/or related cellular abnormalities have been summarized in Table 4 [128,138,140,141,142,143,144,145,146,147]. In this connection, Skeate and his colleagues have given an explanation of the potential mechanisms [148]. It has been demonstrated that secretory leukocyte protease inhibitor (SLPI) prevents HPV-16 entry into cells and subsequent infection. Of note, SLPI, an antimicrobial substance, is produced by a number of cells, including epithelial cells, and is found in a variety of body secretions, including cervical mucus. Although HPV’s cell entry is not understood precisely; in a nutshell, the binding of L1 with the basement membrane heparan sulfate proteoglycans and subsequent interactions with a number of biomolecules, including integrins and growth factor receptors leads to the interaction between the virion and annexin A2 heterotetramer (A2t), which facilitates the viral endocytosis. Interestingly, SLPI inhibits this interaction of A2t and viral particles. However, HSV can downregulate SLPI. By in vitro experiments on human keratinocytes, Skeate et al. showed that in an autocrine/paracrine manner, endogenously secreted SLPI interacted with A2t [148]. Finally, they have concluded that local downregulation of SLPI levels by HSV infection causes enhanced susceptibility to HPV infection via A2t, which may suggest an important role of HSV in HPV-associated tumorigenesis.

Regarding the contradictory behavior of HSV-2 in cervical cancer or relevant epithelial abnormalities, the ‘hit-and-run’ mechanism has been proposed, which suggests that the involvement of HSV-2 is required only in the initial phase of pathogenesis, and its presence is not necessary thereafter [140,146,147]. Nevertheless, seropositivity of HSV-2 does not indicate viral reactivation or active phase. People with HSV-2 can lead a normal life without reactivation/outbreak for a long time. On the other hand, reactivation can cause local inflammation, and if it occurs repeatedly in quick succession, the inflammation may continue along with its sequelae. Unfavorable microorganisms in the cervicovaginal environment perhaps create chronic low-grade inflammation, which could support neoplastic processes.

## 7. Cervical Screening and Associated Complexities

An organized screening system can substantially reduce the incidence and mortality of cervical cancer, and one of the ideal examples is the countrywide active mass screening program of Finland for cervical cancer, which started from 1963 onwards. The screening for cervical cancer seemed to have attained its limit in Finland (the plateau incidence: 4/100,000), and the time to reach this state took about 25 years [149]. In this connection, the decrease in the related mortality in Finland also appeared to be entirely linked to the successful screening program [150]. However, the existing incidence of cervical cancer could be associated with other factors, e.g., immigrant population, smoking, long-term oral contraceptive usage, vaginal dysbiosis including sexually transmitted infections such as infections with *Chlamydia* and *Trichomonas* [150,151,152,153]. Sarkeala et al. noticed in immigrant women that higher educational levels and duration of residence were correlated with a lower risk of cervical cancer, while the risk was raised with rising age at immigration [152]. Since 1992, in Finland, women aged 30–60 years are usually screened every 5 years [150]. Interestingly, Lynge et al. mentioned that in several countries, cervical cancer incidence currently displays a bimodal age distribution with peaks roughly at 45 and 65 years, which is similar to breast cancer [154,155]. Nevertheless, as mentioned earlier, a section of the cervical cancer burden in Finland is connected with the immigrant population. In certain migrant communities, participation in cervical screening has been recorded to be lower compared to the general Finnish women [151,156]. On the other hand, in a study conducted on 879 Finnish unvaccinated women aged 18–75 years between 2014 and 2017, the investigators observed that smoking was associated with an increased frequency of HPV-16 in comparison with other high-risk HPV types [157].

A carefully organized screening program can effectively prevent cervical cancer, but similar effects were not recorded in the incidence of vaginal and vulvar cancer. In fact, the incidences of both these cancers in Finland, along with Denmark and Sweden, remained relatively constant at around 2/100,000 since 1955 [149]. Although vaginal and vulvar cancers are relatively rare malignancies, there are several resemblances among cervical, vaginal, and vulvar cancers. For instance, a substantial number of cases of these three malignancies are linked to HPV and smoking, and they generally belong to the squamous cell type (the most common histological pattern). In this context, a few points may be taken into consideration. Cervical cancer usually starts from the squamocolumnar junction, and there is no similar structure in the vagina or vulvar region. On the other hand, Pulkkinen et al. commented that the number of cervical adenocarcinomas is increasing in high-income countries [158]. As mentioned before, there is possibly a positive association between cervical adenocarcinoma and HPV-negative pathology [30,37]. Nonetheless, the effectiveness of HPV vaccination in regard to vaginal and vulvar lesions has not been properly investigated [159].

Participation in the screening programs in the WHO European Region revealed a very high percentage of tested women in Finland (98.4%), whereas the lowest percentages were documented in nations of the low-income category or with lower levels of female education [160]. It is believed that social factors such as education and awareness play an important role in cervical cancer prevention. Unfortunately, these attributes are not adequate in many countries. Studies from different parts of the world have shown an unsatisfactory awareness of the HPV or cervical cancer problem among young people or the general population. For example, in a recent study in the United States, HPV awareness differed by social factors such as educational attainment and ethnicity [161]. Furthermore, a report documented an overall poor HPV awareness among European adolescents [162]. Similarly, low levels of HPV awareness were recorded in countries like Ghana, Nigeria, Oman, and India [163,164,165,166].

Interestingly, the Indian cervical cancer scenario is somewhat intricate. Initially, cervical cancer was the predominant cancer among Indian women, and it was suggested that the majority of risk/etiological factors were associated with sexual behavior [167,168,169]—although the societies were largely conservative during that period. In the early 2000s, a transition of cancer trend was noticed mainly in urban areas, breast cancer became the most recorded cancer, followed by cervical cancer [170,171,172]. Finally, reports from 2016 clearly revealed the second position of cervical cancer among Indian women, after breast cancer [173,174]. Overall, there has been a declining trend in cervical cancer incidence in India [174,175]. For the last few decades, India has witnessed a process of rapid urbanization and Westernization, which includes changes in lifestyle and sexual behavior. The recent findings of the National Family and Health Survey 2019–21 (NFHS-5) have shown that Indian women, on average, have a roughly similar number of sexual partners compared with men; women in some Indian states have more sexual partners than men. On the other hand, different investigators from India have observed several sexual behavior-related problems, e.g., the involvement of a considerable number of young people in premarital sex, lack of knowledge about safe sex practices, and an upswing in sexually transmitted viral diseases—particularly genital herpes [176,177,178,179,180]. Besides the common people’s poor knowledge about cervical cancer and very limited vaccination against HPV, a poor cervical cancer screening program/attitude in India has been noticed by many investigators [166,181,182,183].

Appropriate educational levels are generally associated with health consciousness, undergoing the screening process or medical check-ups regularly, and getting vaccinated in suitable socioeconomic conditions—in fact, these factors are fairly interlinked. It may be worth noting that an organized screening facility for cervical cancer is known to drastically reduce the prevalence of this disease [93]. For that reason, it is difficult to calculate accurately the exact contributions of the beneficial effects of organized screening programs and the impact of vaccination against HPV. In a proper healthcare system, which is seen in developed countries, both the screening process for cervical cancer and HPV vaccination continue concurrently. Contrarily, the Indian situation is different, and for its declining trend of cervical cancer, some non-specific explanations have been proposed, such as fertility awareness along with delayed first pregnancy and improvement in personal hygiene. Pathological mechanisms connected with the early first pregnancy and multiparity are related to extended cervical tissue injuries that could lead to increased vulnerability to microbial infections, including HPV (Figure 1). However, it is not clear how improvement in general personal hygiene or living conditions can prevent a persistent viral infection in common people’s lives when a considerable number of people do not even know the importance of hand hygiene [184]. Of note, many viruses live in our body asymptomatically; their high prevalence in various populations and highly complex biological mechanisms are poorly understood [8,185]. Interestingly, the non-sexual transmission of HPV, including high-risk HPV, via both vertical and horizontal modes, has been reported [186]. To some extent, this type of infection transmission could be prevented by the improvement in personal hygiene or living conditions.

The declining trend of cervical cancer incidence in India might be linked with an overall declining trend in this disease, which has been observed in many countries worldwide [187,188]. In comparison with different regions of the world, the prevalence of HPV in normal women’s cervical samples is not higher in India [189,190,191,192,193]. In a study conducted by Wagh et al., HPV was found in 7.3% of healthy women (*n* = 2408) [191]; while Dutta et al. recorded 9.9% HPV prevalence after analyzing cervical samples from 2501 women [192]. Another study noticed that 8.4% (110/1300) were positive for HPV [193]. In addition, they found a higher presence of HPV, including high-risk HPV, among young married women. On the other hand, Misra et al. noted an increased rate of squamous intraepithelial lesions with the onset of menopause, and they suggested that the phenomenon could be caused by the gradual reduction in estrogen [194]. In a study from the southern part of India, where samples from 10,580 women (parous or married, aged 30–64) were analyzed, the frequency of squamous intraepithelial lesions was 0.8%, and the mean age at diagnosis was 50.2 years [195]. Although it is evident that an increased percentage of high-risk HPV correlates with the severity of cervical cytological morphology [189,196], the initial phase of pathogenesis is difficult to comprehend. In a study conducted by Moscicki et al., most women with HPV infection did not develop LSIL in a median follow-up of 60 months [197]. Considering the results, the investigators denoted that solely HPV cannot be a sufficient factor for LSIL development. In the same way, Sabeena et al. remarked that due to the ubiquitous nature of HPV, the identification of viral DNA in asymptomatic persons by sensitive analysis alone does not indicate infection [186]. Nevertheless, Moscicki et al. observed distinct risks for HPV and LSIL in their abovementioned study. They also found an association between cigarette smoking and the development of LSIL.

## 8. Smoking and Cervical Cancer Risk

Several studies have recorded that smoking increases the risk of cervical cancer [198,199,200]. Malevolti and her colleagues, in their meta-analysis, revealed the association of smoking with the risk of cervical cancer independently from HPV infection [199]. It is known that different chemicals in tobacco, including combustible tobacco products, are toxic, and many of these compounds have carcinogenic potential, such as nitrosamines, aromatic amines, benzene, and polycyclic aromatic hydrocarbons like benzo[*a*]pyrene. These chemicals may influence the cervical environment in several ways, e.g., HPV replication, promotion of HPV E6 and E7 expression, weakening the immune system, including local immunosuppressive effects, DNA damage, and alteration in cellular gene expression [201]. In an in vitro study using HPV-16-containing SiHa and CaSki cervical cancer cells, Muñoz et al. found that tobacco smoke enhanced the expression of E6 and E7, which finally involved the EGFR/PI3K/Akt/C-Jun signaling pathway activation [202]. These investigators commented that without a suitable cofactor like tobacco smoking, high-risk HPV infection is not a sufficient condition for the development of cervical malignancy, owing to the fact that the majority of such infections are benign and cleared in a natural manner. On the other hand, in a prospective longitudinal study that was conducted in Ireland, cervical smears from 275 HPV-positive women were analyzed [203]. In this study, cervical cells from smokers (*n* = 112) showed a higher frequency of p16/Ki-67 co-expression, and these women exhibited an increased risk for the development of CIN2+ and CIN3+ in comparison with non-smokers.

A recent WHO report (in 2024) displays a continued decreasing trend in tobacco use worldwide. In concordance with this trend, investigators have also shown an overall downward trend of the cervical cancer burden globally [204]. Interestingly, Joseph et al. concluded from their study that occupational exposure to tobacco dust can increase the risk of cervical cancer development [205]. A group of researchers from Greece examined a total of 1540 women, and they found a higher rate of cervical intraepithelial lesions among women who were active smokers or exposed to regular passive smoking and non-smoking women living with partners who smoked [206]. Previously, Brown et al. also observed an association between the quantity of cigarette smoking by the husband and the occurrence of cervical cancer in the wife [207]. Tobacco constituents, including carcinogens, can be transported to the cervical mucus in passive smoking/exposure, or these chemicals can be deposited in the cervical region via seminal fluid [208]. As a consequence, results from different studies have comprehensively demonstrated that secondhand/passive smoke exposure increases the risk of intraepithelial lesions and cervical cancer [209,210,211].

Evidence shows that active smoking could increase the susceptibility to high-risk HPV infection [212,213,214]. Moreover, there is a lower probability of clearing the HPV infection among smokers in comparison with non-smoker HPV-positive women with cytologically normal uterine cervix [215]. Unlike the dispersion of various tobacco-related chemicals in body fluids and their connection to diverse health effects, the transmission of HPVs from an anatomical site to other regions of the body is perhaps doubtful as well as unexpected. For instance, HPV transmission between the oropharynx and uterine cervix may not be possible, and the simultaneous presence of HPV in both sites is rare—although viral characteristics include a strict tropism for stratified squamous epithelial surfaces, which are present in both oropharyngeal and cervical areas [216,217,218]. In addition, tobacco use is an important risk factor for the development of both oropharyngeal and cervical cancers [201,219].

Studies on a substantial number of subjects have revealed that smoking can cause a range of epithelial pathologies from cervical cell abnormalities to CIN3 or worse [220,221]. Among Japanese women who were vaccinated against HPV, Hikari et al. recorded a higher incidence of HSIL in smokers compared to non-smokers [222]. Alternatively, in a retrospective cohort study in the Netherlands, the investigators noticed a higher rate of spontaneous regression of the lesion among non-smokers with CIN2 as compared with smokers [223]. They also documented a better disease regression in nulliparity in comparison to parous women. On the other hand, along with cigarette smoking, the use of hormonal contraceptives has been shown to increase the risk of CIN and cancer development [224,225]. In a cross-sectional study from Mexico, which analyzed the data from cervical tissue samples of 115,651 women, high-risk HPV infection was found to be positively associated with subjects’ smoking habits, number of sexual partners, and the use of hormonal contraceptives [226].

## 9. Hormonal Influences in Cervical Cancer

Although there are inconsistent findings regarding the cancer-promoting role of hormonal contraceptives in the cervix uteri, the overall assessment indicates that the use of oral hormonal contraceptives for more than 5 years can enhance the risk of cervical cancer [227,228]. The reasons for the inconsistent findings could be subjective, such as self-reported descriptions of the study subjects, and/or issues related to hormonal constituents [229]. From a cohort of 308,036 women in the European Prospective Investigation into Cancer and Nutrition (EPIC) Study, with a 9-year median follow-up, the investigators recorded a positive association between oral contraceptive use and the risk of cervical precancerous conditions and cancer [230]. Moreover, they noticed that increased risk was linked with prolonged use, whereas discontinuation of hormonal contraceptives decreased the risk. In the Netherlands, a retrospective population-based cohort study, which included 702,037 women with a median follow-up of 9.7 years, revealed that the use of oral contraceptives or intrauterine device (IUD—copper or hormone-releasing) increased the risk of developing CIN3+ [231]. In addition, the study documented a higher risk of developing CIN3+/cervical cancer among oral contraceptive users in comparison to IUD users. Similarly, a Danish population-based cohort study, which included users of hormone-releasing IUD (*n* = 60,551), copper IUD (*n* = 30,303), and oral contraceptives (*n* = 165,627), noted a higher risk of CIN3+ in oral contraceptive users than the users of both copper and hormone-containing IUDs [232]. Another cohort study from Denmark was conducted from 1995 to 2014, and the investigators followed 1,853,542 women; they found 3643 incident cervical cancer cases [233]. The study displayed an increased cancer risk in current or recent users of any hormonal contraceptives. Many researchers believe that for the development of cervical cancer, along with persistent HPV infection, there is a necessity for some kind of cofactors, e.g., smoking, long-term hormonal contraceptive use, higher parity, and sexually transmitted infections [227,230].

Since oral contraceptives contain estrogen and synthetic progesterone (i.e., steroid hormones) and the uterine cervix is a steroid hormone-sensitive tissue, investigators from both clinical and experimental set-ups showed an involvement of estrogen-related signaling in carcinogenesis. In a study in Switzerland, the investigators observed that higher salivary estradiol levels were positively associated with the presence and persistence of high-risk HPV in a group of nulliparous women (18–31 years) who used combined oral contraceptives regularly in comparison to HPV-negative women within the same group [234]. A population-based cohort study in Taiwan followed 42,940 breast cancer patients with and without antiestrogen therapy [235]. In that study, a lower risk of cervical neoplasia was found in aromatase inhibitor users. A recent case report on an 84-year-old woman with cervical squamous cell carcinoma showed remission after using tamoxifen and, subsequently, letrozole (aromatase inhibitor) [236]. The patient also had diabetes, hypertension, and chronic renal failure. For a long time, various studies revealed a declining trend of estrogen receptors (ER) along with cervical neoplastic progression [237,238]. Of note, estrogen can function through its different receptors, i.e., ERα, ERβ, and G protein-coupled estrogen receptor (GPER/GPER1/GPR30). Another report from Taiwan showed that ERα was primarily expressed in the stroma but was either poorly or not expressed in cancer tissue [239]. Moreover, this stromal ERα expression was lower in advanced disease and thus correlated with prognosis in cervical squamous cell carcinoma. A study from the United States analyzed different cervical samples, i.e., normal specimens, intraepithelial neoplasia (CIN1, 2, and 3), and cancer [240]. The study observed a drop in ERα expression in CIN and cancer cells; although the expression of ERα in stromal cells persisted during the development of cancer, which indicates that estrogen signaling occurs indirectly in cervical cancer via ERα-positive tumor-associated stromal cells, unlike hormone-sensitive breast cancer cells that express ERα directly. Furthermore, in contrast to hormone-sensitive breast cancer, the investigators of this study mentioned the presence of increased interleukin-8 (IL-8) and the absence of detectably expressed ERβ in the cervical tumor environment. However, regarding the overall ER expression pattern, a research group from Mexico documented a similar declining trend of ERα in cervical carcinogenesis, while ERβ expression was maintained [241]. Contrary to the situation of squamous cell carcinoma of the cervix, a study on cervical adenocarcinoma cases (stages Ib-IIa) reported that the expression of ERα was significantly higher in cancer when compared to chronic cervicitis and normal cervix samples [242]. This study also found an association between poor prognosis in cervical adenocarcinoma patients and ERα expression.

In a study in Taiwan, tumor and stromal components from 169 patients with cervical cancer were assessed for the expression of ERα and progesterone receptors (PR), particularly PRB [243]. The investigators of this study observed that the expressions of both ERα and PR were primarily detected in the stromal compartment. Furthermore, stromal PRB expression significantly correlated with a favorable prognosis. Another study that analyzed cervical squamous cell carcinoma tissue samples found that the expression level of *CYP19A1* was upregulated and *ESR1* was downregulated in cancer [244]. It is notable that the encoding genes for ERα and aromatase (which converts androgens to estrogens) are *ESR1* and *CYP19A1*, respectively. On the other hand, a study from Austria conducted immunostaining of tumor core tissue microarray for 126 cervical cancer patients [245]. The study noticed a correlation between ER expression and phosphorylated-mTOR in tumor samples. The investigators concluded that ER expression could be connected with the EGFR/PTEN/mTOR pathway from their findings.

In cervical cancer pathology, Li and Zheng tried to evaluate the role of estrogen-related receptor β (ESRRB), which is a member of the orphan nuclear receptor family and can bind to estrogen response element (ERE) [246]. From this perspective, they examined human tissue specimens (HSIL, cervical carcinoma, and normal cervix tissues), cervical cancer cell lines (HeLa, SiHa, CaSki, C-33A, and HT-3), and an in vivo model (BALB/c nude female mice). Of note, HeLa cells are HPV-18-positive and derived from adenocarcinoma, HT-3 is an epithelial-like cell line with HPV-30 DNA in its genome, and C-33A is an epithelial cell line with elevated expression of p53 and devoid of HPV DNA. Nonetheless, this study revealed that ESRRB was overexpressed in cervical cancer and linked with disease progression, whereas ESRRB knockout in cervical cancer cells triggered cell-cycle arrest, causing interference of cell proliferation in vitro and diminished tumor growth in vivo. In an in vitro experiment with SiHa cervical cells, it has been recorded that a steroid compound with antiestrogenic properties, 13α-estrone sulfamate derivative (13AES3), displayed proapoptotic, anti-migratory, and anti-invasive effects [247]. On the other hand, synthetic estradiol analog 17β-aminoestrogen (17β-AE) compound prolame induced cell proliferation in cervical cancer SiHa cells, but another 17β-AE butolame decreased cell viability [248]. The investigators suggested that the effects of 17β-AEs could be mediated by their interaction with GPER.

The role of GPER in cervical cancer appears to be controversial, owing to reports of both its tumor-inhibiting and growth-stimulatory effects. In an initial study from Germany, cervical cancer tissue samples were analyzed by immunostaining from 156 patients (between 1993 and 2002) [249]. GPER expressions at different cellular compartments were as follows: positive staining at the cell membrane—in 114 (73.1%) cases, cytoplasm—in 129 (82.7%) cases, and both membrane and cytoplasm—in 101 (64.7%) cases. GPER immunostaining was negative in 14 (9.0%) cases. Cytoplasmic GPER expression showed a favorable prognosis for early-stage cervical cancer. Subsequently, a Mexican study examined both cervical cancer tissue sections (*n* = 44, GPER immunohistochemistry) and cervical cancer cell lines (the effect of GPER agonist G1 on SiHa, HeLa, and C-33A) [250]. The investigators observed that immunohistochemically, GPER was commonly present in cervical cancer and expressed mainly in the cytoplasm. In addition, G1 inhibited proliferation and induced apoptosis in the three examined cell lines. A recent report also has demonstrated a migration inhibitory effect of G1 in SiHa and C-33A cells [251]. In a similar fashion, in SiHa cervical cancer cells, G1 has been shown to prevent proliferation, activate apoptosis, and inhibit cell migration [252].

A study from Japan reported that GPER was mainly expressed in endocervical glands, while ERα was expressed in the squamous epithelium at higher levels [253]. For experiments, the investigators used five cell lines: squamous carcinoma cell lines ME-180 (HPV-68 and ERα-positive) and CaSki, adenocarcinoma cell lines HCA-1 (ERα-positive) and HeLa, along with normal endocervical columnar cell line NCC16-P11 immortalized by HPV-16. They noticed a proliferation stimulatory role of estrogen in adenocarcinoma cells and NCC16-P11 cells via GRER, which eventually increased genomic instability. On the other hand, a study investigated the effects of G15 (a GPER inhibitor) in HeLa and SiHa cells [254]. Interestingly, these cancer cells’ key characteristics, such as proliferation, invasion, and migration, were reduced by G15 treatment. In these cells, G15 treatment altered important intracellular molecules, e.g., enhanced the expression of cell adhesion protein E-cadherin and pro-apoptotic Bax, whereas the expressions of anti-apoptotic Bcl-2, and signaling molecules such as phospho-PI3K and phospho-Akt were decreased. The study also detected the presence of GPER in the membrane and cytoplasm of SiHa and HeLa cells [254]. In another study, Ruckriegl and her colleagues conducted transient GPER silencing in HeLa, C-33A, and SiHa cells using RNA interference (RNAi) [255]. Although there were variations in cellular outcome among these three cell lines in response to GPER knockdown, such as a higher number of colonies in HeLa and an expanded size of colonies in C-33A cells, overall, their study exhibited a tumor suppressive function of GPER.

A variant of ERα is ERα36, which has been shown to be expressed in both cervical squamous cell carcinoma and adenocarcinoma tissues, as well as different cervical cancer cell lines [256,257]. Immunohistochemical staining for ERα36 was primarily localized in the cell membrane and cytoplasm of cancer cells. Experiments on CaSki cells and HeLa cells displayed that ERα36 can promote cell proliferation, invasion, and migration capabilities. In concordance with this outcome, Wang et al. documented that overexpression of ERα36 in cervical cancer was associated with poor prognosis [256]. Interestingly, Zhang et al. commented that there is an antagonism between the functions of ERα and ERα36 (stimulatory role) on the expression of HPV E6 and E7, which could be enhanced in a situation of ERα deficiency and ERα36 overexpression that ultimately leads to tumorigenesis [257]. Like ERα36, GPER (mentioned above, another alternative receptor for estrogen) perhaps behaves somewhat similarly, for instance, in cellular locations and signaling via downstream kinase activation involving mitogen-activated protein kinase (MAPK). In a study in Japan, Ino et al. examined 53 cases of cervical adenocarcinoma and 10 cases of adenocarcinoma in situ [258]. They observed a negative correlation between the immunoreactivity of GPER (cytoplasm and membrane staining) and ER (nuclear staining) in the lesions and poor prognosis among patients with GPER overexpression, similar to the role of ERα36.

Several in vivo studies in transgenic mice expressing HPV oncogenes, such as K14E6/E7 and K14HPV16 mice, have elucidated the role of estrogen in cervical cancer [259,260,261]. In this biological environment, estrogen has been shown to promote cervical tumorigenesis via stromal ERα and, in conjunction with HPV oncogenes, alter the expression of various genes, including pro-inflammatory genes, in cervical stromal and epithelial compartments. It may be worth mentioning that the Hedgehog signaling pathway is perhaps important in the coordination of viral and estrogenic pathological processes [262,263]. In this context, De Nola et al. have enumerated a number of involved biomolecules, e.g., heparin-binding EGF-like growth factor, IL-6, matrix metalloproteinase-9, monocyte chemoattractant protein-1, and vascular endothelial growth factor [262]. Nevertheless, they have also suggested a key role of stromal ERα in the release of many of the abovementioned substances in the uterine cervix.

On the other hand, in a recent study, the investigators experimentally infected the reproductive tract of female K17 knockout (K17KO) and wild-type mice (FVB/N genetic background) with MmuPV1 murine papillomavirus [264]. Mice were either untreated or administered exogenous estradiol for six months. It is worth mentioning that an inflammatory condition can induce the expression of stress keratin 17 (K17), which facilitates local immunosuppression in the presence of papillomavirus infection. Therefore, factors such as K17 expression, MmuPV1 infection, and the downregulation of immune components or immunosuppression perhaps work synergistically. In this study, the investigators observed that roughly 30% of wild-type mice and 78% of the K17KO mice spontaneously cleared MmuPV1 infection, and estrogen-treated mice developed persistent infections during a six-month period. The study concluded that estrogen and K17 supported the persistence of papillomavirus infections, at least in part by creating an immunosuppressive state.

## 10. The Role of Vaginal Bacterial Population

Anatomically, the vagina and ectocervix (or exocervix) of the female genital tract are connected to the external environment. So, diverse and large microbial populations are present in this space in normal situations as well as in various disease conditions. Different microbial agents can immensely influence the biochemical characteristics of the cervicovaginal environment. Therefore, on the one hand, this environment might affect the process of normal conception and healthy pregnancy, but on the other hand, it could increase the risk of a number of health problems, including sexually transmitted diseases. Compared to the traditional culture-based isolation of bacteria and other microbial agents, the utilization of various PCR-based techniques and other modern methods has expanded our knowledge of bacterial diversity in the cervicovaginal region. It may be worth mentioning that the cervicovaginal region is highly influenced by various hormonal and other factors, e.g., female reproductive age, oral contraceptives or estrogen replacement therapy, menopausal phase, diabetes mellitus, urinary tract infection, use of systemic corticosteroid drugs or antibiotics, and sexual behaviors [265]. In general, the normal vaginal flora is predominantly maintained by *Lactobacillus* species, particularly *L. crispatus*. *Lactobacilli* are Gram-positive, non-spore-forming, rod-shaped bacteria. It is thought that *Lactobacilli* support the cervicovaginal environment by releasing lactic acid (lower pH), hydrogen peroxide, and antimicrobial substances, which inhibit the colonization of pathogenic microbial agents.

Bacterial vaginosis is a condition when a considerable number of normal flora bacteria of the vagina are replaced by pathogenic organisms that include *Gardnerella vaginalis* (facultative anaerobic bacterium), *Mycoplasma hominis* (opportunistic bacterium that lacks a cell wall), *Prevotella* species (anaerobic Gram-negative bacteria), *Mobiluncus* species (anaerobic bacilli), and certain *Peptostreptococcus* species (anaerobic Gram-positive cocci). Furthermore, investigators noticed that women with bacterial vaginosis were at an increased risk of acquiring *Trichomonas vaginalis* (protozoal) infection [266], and there could be the coexistence of both bacterial vaginosis and vaginal candidiasis (*Candida albicans* fungal infection) [267]. According to CDC, bacterial vaginosis is the most frequent vaginal disease among women of 15–44 years age group, and a report published in 2007 revealed that the estimated prevalence in the United States was about 21 million (29.2%) women [268]. The disease is characterized by foul-smelling, thin, white or greenish vaginal discharge, which is accompanied by vaginal itching and a burning sensation during urination. Interestingly, a meta-analysis suggested a connection between bacterial vaginosis and CIN (i.e., precancerous lesions) [269]. In addition, a study from Brazil documented that certain bacterial vaginosis pathogens, such as *G. vaginalis* and *Megasphaera* species Type 1, could play a critical role in HPV-mediated cervical cancer [270]. On the other hand, Anton et al. observed that bacteria-secreted factors played an important role in the functional modification of cervical epithelial cells, and a state of dysbiosis (i.e., loss of beneficial bacteria) in the cervicovaginal environment could lead to a disturbance of the cervical epithelial barrier [271]. In their study, *L. crispatus* was found to be associated with a favorable outcome in the cervicovaginal space, which protected the cervical epithelial barrier against infections. Therefore, the presence of unfavorable vaginal microorganisms (microbiota) in women could be associated with several obstetrical and gynecological disorders, including cervical cancer (Table 5) [272,273,274,275,276,277,278,279,280,281,282,283,284,285,286]; and a comprehensive understanding of the vaginal bacterial population perhaps is useful in the prevention of many of these problems.

### 10.1. Lactobacillus Species in Vaginal Bacterial Flora

In the majority of healthy women, *Lactobacilli* are the most commonly isolated vaginal bacteria. Taxonomically complex *Lactobacilli* are composed of numerous species (more than 170) that normally inhabit the gastrointestinal tract and cervicovaginal area [287]. In the gastrointestinal tract, common *Lactobacilli* include *L. oris*, *L. salivarius*, *L. rhamnosus*, *L. casei*, *L. fermentum*, *L. helveticus*, *L. mucosae*, and *L. reuteri*. Many of these *Lactobacillus* species are probiotic strains and are utilized in the industrial fermentation of milk. In addition, they can be isolated from the human vagina. However, in the vagina, commonly isolated *Lactobacillus* species are *L. crispatus*, *L. iners*, *L. jensenii*, and *L. gasseri* [288]. It may be worth mentioning that phenotypically different *Lactobacillus* species are not easily distinguished; frequently, molecular identification is required, and studies utilize 16S ribosomal RNA (rRNA) gene sequencing (Table 6, [289,290,291,292,293,294,295,296,297,298,299]).

**Figure 3:**
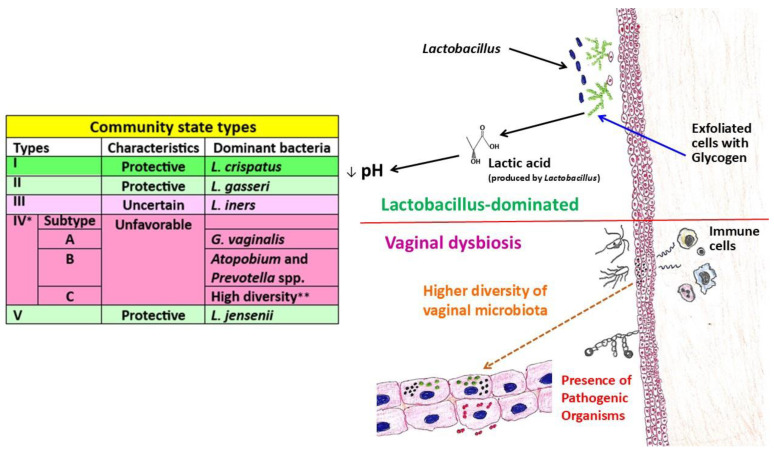
Shows vaginal bacterial flora and dynamic interactions between the community state types (particularly community state type-III and community state type-IV) that are associated with vaginal dysbiosis and pathological conditions. * Several bacteria with high pathogenic potential, e.g., *Prevotella*, *G. vaginalis*, and *Atopobium vaginae* are linked with biofilm formation. ** Heterogenous bacteria such as *Corynebacterium*, *Bifidobacterium*, *Streptococcus*, *Ureaplasma*, *Escherichia*, and *Peptostreptococcus* [302].

Depending on the presence of specific *Lactobacillus* species and their quantity along with the relative abundance of other microorganisms, vaginal microbial flora can be subdivided into five (or sometimes more) bacterial community groups or community state types [300,301,302]. In brief, the general characteristics of these community state types are as follows (Figure 3):

(A) Community state type-I: This is a common type, dominated by *L. crispatus*, and linked with a healthy condition of microbial flora. In general, this type is observed in white women. Furthermore, this type has been characterized by a low pH environment (lower than 4.0), a relatively stable state, and its transition to a pathologic situation is rare. However, during a number of physiological conditions, such as sexual practice, menstrual periods, and pregnancy, there could be a temporary transition to a state wherein *L. iners* or mixed species might dominate.

(B) Community state type-II: This type is rarely found, and *L. gasseri* dominates it. Interestingly, this type maintains an environment of low vaginal pH (around 4.4). However, during pregnancy, rarely, the transition to the community state type-I may occur, while the transition to any pathological state is rare.

(C) Community state type-III: This type is commonly observed and dominated by *L. iners*. Unlike the abovementioned two types, it is characterized by the decreased glycolytic enzymatic activity and the resultant higher vaginal pH (more than 4.5), which is not sufficient to inhibit the growth of pathogens. Consequently, this type is associated with higher levels of pro-inflammatory cytokines, e.g., IL-1α, IL-18, tumor necrosis factor-alpha, and macrophage migration inhibitory factor. In addition, from this type, the transition to the community state type-IV can occur more frequently.

(D) Community state type-IV: Overall, this type is commonly found in healthy African black women. Moreover, this type has been further categorized into two or three subgroups. Subgroup IV-A can be distinguished by *G. vaginalis*-dominated or moderate quantities of *L. crispatus*, *L. iners*, or other *Lactobacillus* species with low proportions of anaerobic bacteria such as *Anaerococcus*, *Corynebacterium*, *Finegoldia*, or *Streptococcus*, as well as low Nugent scores [300,303]. Of note, the Nugent criteria/scores assess the proportions between *Lactobacillus* species and bacterial vaginosis-associated pathogens (from vaginal swabs and by Gram-stain) in order to construct a scale from normal to disease state [304]. Higher scores (i.e., 7–10) indicate bacterial vaginosis. On the other hand, in subgroup IV-B, greater proportions of *Atopobium* have been identified along with other bacterial species, e.g., *Prevotella*, *Parvimonas*, *Sneathia*, *Gardnerella*, *Mobiluncus*, or *Peptoniphilus* [300]. This situation has been commonly detected in patients with bacterial vaginosis. A lower number of *Lactobacilli* cannot produce a sufficient amount of lactic acid (i.e., higher pH), and thus, disturbance of the mucin layer by bacterial vaginosis-associated bacteria (BVAB) reduces the adhesion of *Lactobacilli*, which are not capable of preserving the mucosal layer. It is worth mentioning that BVAB have been determined by the molecular characterization that has displayed three uncultured species, i.e., BVAB1, BVAB2, and BVAB3, belonging to the order *Clostridiales*. Nevertheless, in bacterial vaginosis patients, higher Nugent score and biofilm formation by BVAB in vaginal epithelium have been observed. Moreover, this subgroup has been shown to be associated with increased levels of pro-inflammatory cytokines, as well as an elevated risk of sexually transmitted infections, pelvic inflammatory diseases, preterm labor, and miscarriage. With regard to this subgroup, the transition to the community state type-III can happen, but rarely to the community state type-I.

(E) Community state type-V: This type is dominated by *L. jensenii* and is rarely found. Nonetheless, this is a stable type, with a vaginal pH 4.2 (average). Although available reports are not enough, the transition to other community state types may not happen.

(F) Type that is not classified: This type includes a number of *Lactobacillus* species, e.g., *L. fermentum*, *L. helveticus*, *L. mucosae*, *L. oris*, *L. reuteri*, *L. rhamnosus*, and *L. salivarius*. Many of them are associated with the gastrointestinal tract. This group has not been placed in any community state type(s) because of inadequate information. These *Lactobacilli* are primarily isolated from asymptomatic Indian women, with low vaginal pH (lower than 4.5, similar to other healthy community state types).

It is clear that the abovementioned four community state types (out of five) are *Lactobacillus*-dominated; and *L. crispatus* and *L. iners* are common vaginal inhabitants. Although these two *Lactobacillus* species are very similar, surprisingly, they are rarely noticed to coexist for a prolonged period [305]. As compared with *L. crispatus*, the *L. iners* genomic size is considerably shorter (approximately half). The reduced genomic size of *L. iners* restricts its metabolic abilities and increases its dependence on external resources. For this reason, *L. iners* is more susceptible to environmental fluctuations, and *L. iners*-dominated flora may create an unstable environment [305]. Typically, the domination of *L. crispatus* in community state type-I is an indication of a healthy cervicovaginal space. On the other hand, *L. iners*-dominated community state type-III has been considered to be associated with vaginal dysbiosis and infection with various pathogens (Figure 3) [271].

### 10.2. Diminished Vaginal Lactobacillus Dominance and Cervical Lesions

**Figure 4:**
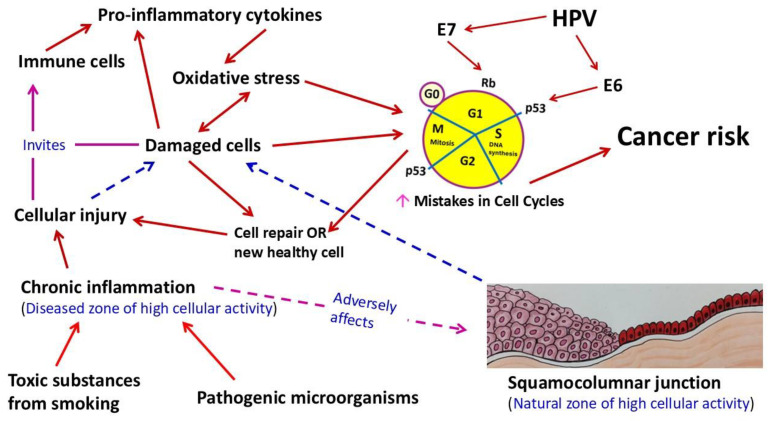
Possible role of co-infections in cervical cancer risk. Cervical cancer usually originates from the squamocolumnar junction. E6 and E7 of HPV inactivate the p53 and retinoblastoma proteins (Rb), respectively.

Several studies have observed that vaginal dysbiosis was associated with HPV infection and cervical cancer [306,307,308]. This dysbiosis is clearly linked with the alterations of favorable *Lactobacillus* species. Broadly, different clinical studies have observed a decline in *Lactobacillus* dominance, particularly *L. crispatus*, and an increased proportion of certain bacteria, such as *Gardnerella*, *Prevotella*, and *L. iners* (Table 7, [309,310,311,312,313,314,315,316,317,318,319]). A recent meta-analysis based on 36 studies published between 2014 and 2023 also documented similar findings [320]. Vaginal dysbiosis and the accompanying diminished *Lactobacillus* population could be responsible for chronic low-grade inflammation, epithelial injury, and persistent HPV infection (Figure 4). It is well known that chronic low-grade inflammation increases the risk of various cancers, which can also be noticed in obesity-related cancers due to the imbalanced or excessive release of pro-inflammatory adipokines [321]. In connection with dysbiosis and the diminution of beneficial *Lactobacillus*, several authors have pointed out a number of pathological phenomena, e.g., breach of the epithelial barrier, chronic inflammation, cytokine imbalance, genomic instability, altered immune responses, and dysregulation of estrogenic effects [322,323,324,325]. Obviously, these tissue alterations could lead to malignant transformation.

Norenhag et al. concluded that, compared with *L. crispatus*, the domination of *L. iners* or other non-*Lactobacillus* species in the vaginal flora can increase the risk of HPV infection, cervical dysplasia, and cancer [326]. A study conducted by Chao et al. on 86 HPV-negative and 65 HPV-positive healthy women revealed that in the vaginal flora, anaerobic bacteria such as *Bacteroides plebeius*, *Acinetobacter lwoffii*, and *Prevotella buccae* were detected more frequently among HPV-positive women [327]. In a similar manner, Qingqing et al. found that vaginal dysbiosis, particularly higher presence of *Anaerococcus*, *Prevotella*, and *Sphingomonas*, correlated with persistent HPV infection [328]. In a recent report, Leon-Gomez and Romero explained how HPV persistence is linked with vaginal dysbiosis marked by higher microbial diversity, increased anaerobic bacteria, and reduced *Lactobacillus* species [329]. Here, it may be worth mentioning that in an in vitro study, supernatants of three *Lactobacillus* species (*L. crispatus*, *L. jensenii*, and *L. gasseri*) exhibited inhibitory effects on the viability of CaSki cervical cancer cells [330].

Considering the importance of *Lactobacillus*, a study was conducted by intravaginal transplantation of a natural probiotic strain, *L. crispatus* chen-01, in women with high-risk HPV infection [331]. After 6 months, cervical exfoliated cells were collected to examine the status of HPV and cytology. Interestingly, it was observed that vaginal transplantation with *L. crispatus* chen-01 significantly decreased HPV load, enhanced HPV clearance, and ameliorated the state of vaginal inflammation, without any obvious adverse reactions. In a study in Korea, patients with CIN3 and HPV-16 infection were treated with oral capsules, which contained *L. casei* that expressed HPV E7 protein on their surface [332]. Subsequently, 16 weeks after the initial treatment, six patients (out of eight) experienced clinical effectiveness: remission to normal in three, and to CIN1 in three cases. In addition, there is some evidence that shows a connection between gastrointestinal and vaginal microbiota [333,334,335]. For this reason, the gut-vaginal axis has been hypothesized, which could be an interesting concept for novel pharmaceutical strategies.

The vaginal microorganisms survive in a highly complex and fluctuating microenvironment, which is continually affected by various events, e.g., menstrual periods, sexual practice, pregnancy, estrogenic status, vaginal douching, and treatment with antimicrobial agents or systemic corticosteroid therapy. The native or original vaginal microorganisms are thought to exist in a symbiotic/ecological association with the host. Several studies have shown the beneficial role of *Lactobacillus* species, especially *L. crispatus*, in the health and prevention of a number of gynecological diseases, including neoplastic transformation. Strategies for successful vaginal *L. crispatus* transplantation from healthy donors are potential areas of therapeutic research for women at risk.

## 11. Conclusions

Globally, the majority of people acquire HPV in some body parts that are communicated with the exterior environment due to the ubiquitous nature of this virus, but only a few women finally develop cervical cancer. Furthermore, in a particular person, a specific HPV genotype may not be present constantly; alterations in HPV genotypes can occur [49]. Interestingly, an inverse association was found between β/γ-HPV and CIN [111], although high-risk HPVs belong to the α genus. Therefore, there are numerous intricacies. However, several scientists believe that HPV alone cannot cause the malignant transformation in the cervix uteri. For cancer development, presumably, there is a need for the involvement of other factors such as smoking, prolonged use of hormonal contraceptives, the number of sexual partners, and dominance of unfavorable bacteria in the cervicovaginal environment. This is a promising aspect because the pathogenic pathways of cancer development can be blocked by suitably altering or impeding the abovementioned factors. Currently, a continuously decreasing trend in the incidence of cervical cancer has been noticed among certain unvaccinated populations. This interesting phenomenon is likely connected to educational attainment and a significant reduction in smoking habits. At an individual level, a proper education modifies several facets of one’s life, such as the development of logical reasoning, lifestyle changes, a smaller household size, and the management of adequate food and nutrition. A recent study also recorded similar observations [315]. The women and their families in large geographical regions live in poverty, and these women with poor socioeconomic status are at the highest risk for cervical cancer. They have no or poor access to health screening and practically no access to vaccination against HPV. Nevertheless, the development of an appropriate education system can reduce several health problems, including cervical cancer risk, through lifestyle modification. On the other hand, the concepts of the gut-vaginal axis and restoration of beneficial *Lactobacillus* dominance are fascinating approaches; adequate studies are needed to develop a low-cost preventive strategy. 

## Figures and Tables

**Table 1:** Selected studies that examined the presence of viruses in neuroendocrine tumor tissues from the uterine cervix (and associated structures), lungs, adrenal glands, and pancreas.

Tumor Type	Authors, Place of Study, and Year of Publication	Study Designs	Findings in Brief
Cervical cancer (neuroendocrine tumors)	Shen et al., 2025 (China) [48]	Neuroendocrine cervical carcinoma samples from eight patients were analyzed	One patient had cancer of mixed types, which was excluded but HPV-16 positive. Out of seven cases, six were HPV-18 positive, and one was positive for HPV-16.
	Park et al., 2025 (Korea) [49]	Case report: Primary malignant melanoma of the vagina from a 53-year-old female patient	The initial diagnosis was atypical squamous cells of undetermined significance, positive for HPV-39, -68, and -74; after 1 year, LSIL, positive for HPV-52 and -58; afterwards, ASC-H, positive for HPV-50 and -51.
	Wei et al., 2024 (China) [50]	Two cases of cervical mixed cancer of large-cell neuroendocrine carcinoma and adenocarcinoma	One case was HPV-18 positive.
	Liu et al., 2024 (China) [51]	Seven cases (out of eleven) with small-cell carcinoma of the cervix were tested for HPV before treatment—between 2017 and 2023	All seven were positive for HPV; five cases were positive for HPV-18 and two cases were HPV-16.
	Gordhandas et al., 2022 (United States) [52]	A total of 63 patients with small-cell neuroendocrine carcinoma of the cervix (41 had limited-stage and 22 had extensive-stage); from January 1990 to June 2021	Overall, HPV positive cases were 17 (34%, limited-stage: 13 or 38%). Missing cases: 13.
	Schultheis et al., 2022 (Germany and United States) [53]	Nine cases of uterine cervix small-cell carcinomas were detected over a 20-year period	Eight cases were HPV-18 and one case was HPV-16 positive.
	Lu et al., 2022 (China) [54]	Evaluation of HPV for 9 cases with small-cell carcinoma (out of 19); from 2012 to 2021	HPV infection rate was 77.77% (7/9); HPV-18: 5, HPV-16: 1, and HPV-52: 1.
	Ordulu et al., 2022 (Multinational study) [55]	14 cases (small-cell carcinoma: 6, large-cell carcinoma: 6, other neuroendocrine tumors: 2)	Except for three cases of small-cell carcinoma, all cases were positive for HPV.
	Pei et al., 2021 (China) [56]	51 patients with small-cell carcinoma, between 2007 and 2020	In all cases, HPV infections were detected (HPV-18 in 47 cases).
	Van Ta et al., 2019 (Vietnam) [57]	30 neuroendocrine tumors	HPV was identified in 26 (86.7%) cases; HPV-18 in 15 cases.
	Feng et al., 2018 (China) [58]	82 patients with neuroendocrine carcinoma; from 2008 to 2016 (small-cell carcinoma: 74, large-cell carcinoma: 7, atypical carcinoid: 1)	HPV was detected in 72 cases; high-risk types in 70 cases, and HPV-18 in 49 cases.
	Siriaunkgul et al., 2011 (Thailand) [59]	97 cases with neuroendocrine carcinoma; between 1992 and 2009	HPV was detected in 93 samples (95.87%), of which 76 were single HPV type infection; HPV-18: 70 cases.
	Wang et al., 2006 (Taiwan) [60]	26 specimens were analyzed (out of 31 cases, between 1991 and 2003)	In 18 cases, HPV infection was present; HPV-negative cases: 8, HPV-18: 17, and HPV-16: 1.
	Ishida et al., 2004 (Japan) [61]	10 cases of small-cell carcinoma, 3 were pure small-cell cancer	HPV was detected in 7/10; HPV-18 was detected in all pure tumors and four mixed tumors; no other HPV types were isolated.
Small-cell lung cancer and other neuroendocrine tumors of the lung	Han et al., 2024 (China) [62]	36 SNPs from 4 genome-wide association studies’ data were examined with HPV-16 E7 and HPV-18 E7	No significant association was found between small-cell lung carcinoma and HPV-16 E7 or HPV-18 E7.
	Sirera et al., 2022 (Spain) [63]	Specimens were collected from 41 patients with lung cancer, including 3 small-cell carcinoma cases (7%)	Two cases were positive for HPV: one case of HPV-16 infection, and in another case, HPV was not classified (low- or high-risk).
	Zou et al., 2021 (China) [64] *	E6 and E7 mRNA levels of HPV-16 were detected in the bronchial brushing and TBNA of 184 patients with lung cancer (44 cases of small-cell lung cancer) and 126 benign lung diseases	Both E6 and E7 mRNA expression levels in small-cell carcinoma and squamous cell carcinoma (*n* = 80) were significantly higher compared to benign cells. Among small-cell cancer, the expression levels in 38 central type cases were significantly higher than in six cases of peripheral type.
	de Oliveira et al., 2018 (Brazil) [65]	63 samples of lung cancer from different histological types	HPV was found in 33 samples; small-cell carcinoma: 18.18%, and large-cell carcinoma: 9.1%.
	Shikova et al., 2017 (Bulgaria) [66]	132 lung cancer specimens of different histological types including 24 small-cell carcinoma cases were analyzed for HPV	In the small-cell carcinoma group, HPV was present in seven cases (29.2%); HPV-16: two, HPV-18: three, HPV-16 + HPV-18 coinfection: two.
	Hartley et al., 2015 (United States) [67]	19 patients’ specimens; from 2004 to 2013	No HPV was found.
	Castillo et al., 2006 (Mexico, Colombia, and Peru) [68]	36 lung carcinomas of different histological types including 9 cases of small-cell carcinoma	Among small-cell carcinomas, HPV was detected in three cases, which were positive for HPV-16.
	Brouchet et al., 2005 (France) [69]	122 cases of lung cancer, including small-cell carcinoma (*n* = 9), large-cell neuroendocrine carcinoma (*n* = 13), typical carcinoid (*n* = 6), and atypical carcinoid (*n* = 3). In tissue sections, the presence of HPV, EBV, HHV-8, CMV, and SV40 was investigated.	None of the cases showed any positive results.
	Thomas et al., 1996 (France) [70]	31 biopsies of lung cancer (different histological types), including 6 small-cell carcinoma cases	HPV was positive in 1 small-cell carcinoma (1/6) and in 1 other neuroendocrine tumor (1/1).
	Schmitt et al., 2011 (Germany) [71]	Neuroendocrine tumors from various sites, including lungs and skin, were analyzed (*n* = 43) for different viruses (e.g., HPV, EBV, HBV, MCV)	EBV was positive in 1 case (1/6) of pulmonary large cell carcinoma and in 1 case (1/5) of small-cell carcinoma; 2 cases (2/3) of Merkel cell carcinoma were positive for MCV.
Pheochromocytoma	Oraibi et al., 2018 (United States) [72]	Case report: pheochromocytoma with primary adrenal lymphoma	Lymphoma was associated with EBV.
	Badani et al., 2016 (United States) [73]	63 specimens of adrenal gland tissue, including 21 cases of pheochromocytoma, were analyzed for VZV and HSV-1	VZV was found in four normal adrenal gland tissue samples. No VZV or HSV-1 was detected in cases of pheochromocytoma.
	Sathe et al., 2012 (India) [74]	Case report: a pediatric case with bilateral adrenal neoplasms (without immunodeficiency)	Adrenal leiomyoma that was associated with EBV (initially, pheochromocytoma was suspected).
Pancreatic neuroendocrine tumors	Vemuri et al., 2022 (Australia) [75]	Case report: a patient with HIV and HBV coinfection	VIPoma in the pancreatic tail was diagnosed.
	Fiorino et al., 2015 (Italy) [76]	Case report: a patient with cirrhosis and two synchronous malignancies—in the liver (hepatocellular carcinoma) and in the pancreatic tail (neuroendocrine tumor)	In neuroendocrine tumor cells, the HBV genome was detected.

CMV: Cytomegalovirus, EBV: Epstein–Barr virus, HBV: Hepatitis B virus, HHV: Human herpesvirus, HIV: Human immunodeficiency virus, HPV: Human papillomavirus, HSV: Herpes simplex virus, MCV: Merkel cell polyomavirus, SV40: Simian virus 40, VZV: Varicella zoster virus. SNPs: Single-nucleotide polymorphisms, TBNA: Transbronchial needle aspiration. ASC-H: Atypical squamous cells, cannot exclude high-grade squamous intraepithelial lesion (HSIL), Leiomyoma: Benign neoplasm from smooth muscle, LSIL: Low-grade squamous intraepithelial lesion, Merkel cell carcinoma: Neuroendocrine cancer of the skin, Pheochromocytoma: Tumor from chromaffin cells of the adrenal medulla, VIPoma: Vasoactive intestinal peptide-secreting tumor. * According to the investigators’ observation, in increasing frequency, epidemiological and clinical data demonstrate that HPV infection is closely associated with the carcinogenesis of organs, which directly communicate with the outside of the body.

**Table 2:** A simplified grouping of different HPV types.

Classification	HPV Genotypes
High-risk	16, 18, 31, 33, 35, 39, 45, 51, 52, 56, 58, 59, 66, 68
Probable high-risk	26, 30, 34 *, 53, 66, 67, 68, 69, 70, 71, 73, 82, 90
Undetermined	2, 3, 7, 10, 27, 57, 62, 85, 91
Low-risk	6, 11, 32, 40, 42, 43, 44 **, 54, 61, 72, 74, 81, 83, 84, 86, 87, 89

Type: L1 DNA sequence must be different by more than 10% from any other type; Subtype: 2–10% different. * HPV-34 (including subtype 64); ** HPV-44 (including subtype 55). Broadly, HPVs can be categorized into five genera—alpha (α), beta (β), gamma (γ), mu, and nu [85]. In general, high-risk and low-risk HPVs belong to the genus alpha. On the other hand, the most isolated HPVs (~100 types) belong to the genus gamma, while the genera mu and nu comprise only a few HPVs. Excluding a small number of alpha-genus HPV types such as 2, 3, 10, 27, and 28 (which reside in the skin), by and large, the genera beta, gamma, mu, and nu include cutaneous HPV types. Some of these HPVs, e.g., 5 and 8 (of species β1) and 197 (of species γ27), have been thought to be associated with skin carcinogenesis. Of note, in the genus beta, more than 54 HPV types are subdivided into five species, i.e., β1-β5, whereas the genus gamma includes 27 species.

**Table 3:** Recent studies on the status of HPV and HIV that could influence cervical lesions.

Authors and Brief Study Outline	Findings
Lovane et al., 2025 [108]|40 cases of endocervical adenocarcinoma; Mozambique, 2017–2018.	A total of 14 were HIV-positive. Every case was positive for at least one of the HPV-16/-18/-45 genotypes; HPV-18 was the most common type. Multiple infections were exclusively detected in HIV-negative cases.
Debeaudrap et al., 2025 [109]|2253 WLHIV; Côte d’Ivoire, Burkina Faso and Cambodia, 2019–2021.	A total of 932 (41%) were HPV-positive. Of the 777 HPV-positive participants with histopathology results, 105 (13.5%) had CIN2+ lesions, and 75 (9.5%) had CIN3+ lesions.
Pillay et al., 2025 [110]|235 young sexually active women; South Africa	HIV was detected in 49 (20.4%) subjects. HPV DNA was found in 147 (62.6%) urinary and 177 (75.3%) cervico-vaginal lavage samples. HIV positivity is statistically associated with the presence of HPV DNA in urine and cervico-vaginal lavage.
Strickler et al., 2024 [111]|WIHS ongoing cohort (enrolled 2793, CIN2 and CIN3: 124, controls: 247); United States.	In women with HIV, cigarette smoking, parity, and CD4 cell count displayed a positive association with cervical precancer condition. However, there was a strong inverse (protective) relationship between beta-/gamma-HPV infection and the risk of cervical precancer.
Naicker et al., 2024 [112]|260 WLHIV; South Africa.	Approximately 67% of women were positive for high-risk HPV, and about one-third had abnormal cervical cytology.
Mbulawa et al., 2024 [113]|325 participants (HIV-positive: 208); South Africa.	HPV prevalence was 67.8% among HIV-positive women, and HPV infection was found to decrease with increasing age. HPV-58 was the most prevalent type. Among HPV-vaccinated women, 12.9–42.2% (depending on the vaccines) were infected with one or more HPV types.
Rantshabeng et al., 2024 [114]|171 non-HPV-vaccinated indigenous women; Botswana.	A total of 53/171 (31%) and 56/171 (32.7%) were positive for HIV and high-risk HPV, respectively, and 23/171 (13.5%) had cervical dysplasia. In women with cervical dysplasia, commonly detected HPV types were HPV-39, HPV-51, HPV-52, and HPV-56, while HPV-16 and -18 were not found.
Murenzi et al., 2024 [115]|Paraffin-embedded tissue sections from 227 cases (out of 440 cervical carcinomas) with valid HPV results; Tanzania, 2020.	A total of 65 (28.6%) were WLHIV. 19.8% (*n* = 45) were HIV negative, and 55.1% (*n* = 117) were of unknown HIV status. Interestingly, HPV-68 was associated with HIV positivity.
Muchaili et al., 2024 [116]|4612 female subjects screened for HPV infection; Zambia, 2021–2022.	Among the 2734 WLHIV, 1960 were referred to the cancer clinic by the ART facility. Of the participants, 1640 (35.56%) were HPV-positive, of which 986 (36.06%) were also positive for HIV.
Worku et al., 2024 [117]|915 HIV-positive patients at the ART clinic who were not screened for cervical cancer previously; Ethiopia, 2021.	The VIA test showed a positive result in 224 (24.48%) women. Among VIA-positive cases, the diagnostic assessment revealed that 72.4% had abnormal cervical pathology.
Manga et al., 2024 [118]|599 female sex workers aged 30 years and older; Cameroon, 2020.	Overall, 372 (62.1%) were positive for HPV, and 218 were positive for both HPV and HIV. HPV-51 and -53 were the most common types, whereas HPV-18 and -16 were the least prevalent. HIV-positive cases were 1.65 times more likely to be infected with HPV compared to the HIV-negative group.
Akakpo et al., 2023 [119]|330 WLHIV; Ghana, 2020–2021.	In total, 141 women had HIV/HPV co-infection. The most common high-risk HIV type was HPV-59 (50.3%, *n* = 71).
Grover et al., 2023 [120]|1131 cervical cancer patients; Botswana, 2015–2020.	More than 68% (*n* = 770) of these patients were HIV-positive.
Kangethe et al., 2023 [121]|647 WLHIV; Kenya, 2021–2022.	Out of 224 WLHIV with high-risk HPV infections, 21.4% had abnormal cervical cytology; HPV-52 was the most common genotype.
Megersa et al., 2023 [122]|406 WLHIV; Ethiopia, 2022.	More than one-third of WLHIV had high-risk HPV (35.2%, *n* = 173). The commonest type was HPV-16 (15.3%, *n* = 62),
Sørbye et al., 2023 [123]|Subjects *n* = 710: HIV-negative (373/52.5%), WLHIV (337/47.5%); South Africa, 2016–2020.	The HPV positivity rate and CIN3+ prevalence were significantly higher among WLHIV compared to HIV-negative women.
Gupta et al., 2022 [124]|Cross-sectional study: 135 WLHIV and 160 HIV-negative women; India, 2019–2021.	WLHIV had significantly higher cervical cytological abnormalities (14.1%, *n* = 19) and high-risk HPV infection (28.9%, *n* = 39) than HIV-negative women (cytological abnormalities: 3.1%, *n* = 5; HPV: 9.3%, *n* = 15).
Lewis et al., 2022 [125]|1405 women after first-time VIA screening (1305 WLHIV); Malawi, 2017–2019.	Among WLHIV, 65 cases had pre-cancerous lesions (5%), and suspected cancer cases were 13 (1%). In only three HIV-negative women (3%), pre-cancerous lesion was detected. There was no association between the screening outcome and the status of HIV.
Nakisige et al., 2022 [126]|188 WLHIV and 116 HIV-negative women; Uganda, 2017–2020.	High-risk HPV was detected in 67% of WLHIV and 52% of HIV-negative women. CIN3 was present in 41 WLHIV (22%) and 7 HIV-negative women (6%); however cervical cancer was diagnosed in 13 WLHIV (4%) and 24 HIV-negative women (21%). HPV-16 was the most common high-risk type.
Mcharo et al., 2021 [127]|Out of 2134 women, 804 were included (418 WLHIV and 386 HIV-negative); Tanzania, 2013–2020.	CIN1–61 WLHIV and 13 HIV-negative, HSIL (CIN 2 and 3)–52 WLHIV and 16 HIV-negative, cervical cancer–107 WLHIV and 129 HIV-negative. Roughly 80% of CIN1 and HSIL cases were WLHIV.
Jary et al., 2021 [128]|144 women screened for cervical cancer (WLHIV: *n* = 44, high-risk HPV: *n* = 90); Mali, 2018.	High-risk HPV infection was significantly higher in WLHIV compared with HIV-negative women. Moreover, the prevalence of HSV-2 was significantly higher in WLHIV than in HIV-negative women, and in women with high-risk HPV infection compared with those not infected with high-risk HPV.
Taku et al., 2021 [129]|Cross-sectional study: 205 cervical tissue specimens; South Africa, 2017–2018.	High-risk HPV, HIV, and HSV-2 were detected in 66 cases (32.2%), 79 cases (38.5%), and 12 cases (5.9%), respectively. Both high-risk HPV and HSV-2 infections were present in 8 subjects, and 34 subjects had both high-risk HPV and HIV infections. HIV and HSV-2 infections were significantly and positively associated with high-risk HPV infection.

ART: antiretroviral therapy, CIN3+: cervical intraepithelial neoplasia grade 3 or higher/cancer, HSIL: high-grade squamous intraepithelial lesion, HSV-2: Herpes simplex virus type 2, VIA: visual inspection with acetic acid, WIHS: the Women’s Interagency HIV Study, WLHIV: women living with HIV.

**Table 4:** Studies that recorded a positive correlation between HSV-2 and cervical cancer, including cytological abnormalities, in comparison with the findings that did not demonstrate any such relationships.

Positive Correlation	No Correlation
Smith et al., 2002 [138]. Samples were collected from seven countries. Cervical exfoliated cells were collected from all subjects, and biopsy specimens from cancer patients (1158 squamous cell carcinoma and 105 adenocarcinoma or adenosquamous carcinoma). HSV-2 seroprevalence was significantly higher (*p* < 0.001) among cancer patients than control subjects. Similar significance was observed among the HPV DNA-positive women. Among patients (total- 1263), 560 were HSV-2 seropositive, and among control subjects (*n* = 1117), 286 were HSV-2 positive. Cervical samples from 1098 (94.8%) squamous cell carcinoma, 95 (90.5%) adenocarcinoma or adenosquamous carcinoma, and 164 (14.7%) controls were positive for HPV DNA.	El-All et al., 2007 [141]. The study was conducted in the Egyptian population and included complete data from 5453 women. Abnormal changes in the cervical epithelium were found in 424 women. Among them, invasive carcinoma was diagnosed in four cases. Other abnormalities are: low-grade lesions (41.0%), high-grade lesions (5.2%), atypical glandular cell of undetermined significance (15.3%), and atypical squamous cells of undetermined significance (ASCUS) (34.4%). Only one case from the latter category was positive for HSV-2. HPV was evaluated in 217 cases; 66% were positive, 29% were negative, and 5% had no definite diagnosis.
Paba et al., 2008 [142]. In this Italian study, 149 cervical cancer and CIN biopsies were examined. HPV DNA-positive cases were 136, *Chlamydia trachomatis* DNA was identified in 32 cases, and 29 were positive for HSV-2. *C. trachomatis* was significantly associated with multiple-type HPV infections (*p* = 0.0001). Whereas, there was a borderline significance (*p* = 0.053) for HSV-2 infection. Survivin, a member of the family of inhibitor-of-apoptosis proteins, was overexpressed in lesions that were positive for both HSV-2 and HPV (*p* = 0.027).	Wohlmeister et al., 2016 [143]. In this cross-sectional study in Brazil, cervical cytological samples were collected from 169 women during routine gynecologic examination. No cellular abnormalities were found in 75 women (HPV-positive: 9), reactive or inflammatory benign features were seen in 76 women (HPV-positive: 8), and atypia or cervical lesions were diagnosed in 18 cases (all were HPV-positive), which included 10 low-grade lesions, 3 high-grade lesions, and 4 cases of ASCUS. Only one subject was positive for HSV-2.
Kwaśniewska et al., 2009 [144]. This study was conducted in Poland, and the study groups consisted of 520 patients with squamous cell carcinoma of the cervix (HPV-positive: 468, 90%), 50 adenocarcinomas, and 50 controls. In patients with squamous cell carcinoma, HSV-2 was detected in 145 cases (28%) and *C. trachomatis* in 135 cases (26%), and in adenocarcinoma, HSV-2 was detected in 15 cases (30%) and *C. trachomatis* in 12 cases (24%); 4 controls were positive for HSV-2 as well as for *C. trachomatis*. Compared to the control group, a significantly higher occurrence of HSV-2 and *C. trachomatis* was noticed in specimens from patients with cervical cancer (*p* < 0.05). No correlation was seen between HPV and HSV-2.	Moharreri et al., 2021 [145]. The study was performed on 195 liquid-based cytology specimens collected from Iranian women; 50 samples were from CIN cases. Finally, 148 HPV-positive samples were analyzed for different sexually transmitted pathogens such as *Mycoplasma genitalium*, *C. trachomatis*, and HSV-2. *C. trachomatis* infection was present in 24 cases, *M. genitalium* infection in 3 cases, and only 1 case was positive for HSV-2. No statistically significant differences were found between these pathogens and CIN.
de Abreu et al., 2016 [146]. A total of 838 women were enrolled from basic health units of the Brazilian public health system for cervical screening. Among them, 614 women had no epithelial lesions (HPV: 101, high-risk HPV: 66), and 224 women had the following cervical abnormalities (HPV: 183, high-risk HPV: 164): 56 low-grade lesions, 71 high-grade lesions, 65 ASCUS, 27 atypical squamous cells- cannot exclude high-grade squamous intraepithelial lesion, and 5 atypical glandular cells. HSV-2 infection was present in 20 subjects who had no epithelial abnormalities, and in 13 cases who had cervical abnormalities. The coexistence of HPV-DNA and high-risk HPV infection with HSV-2 showed an elevated risk of ≥ ASCUS cytology, but no enhanced risk of high-grade lesions was noticed.	Jary et al., 2021 [128]. In this study in Mali, women infected with HIV (*n* = 44) and HIV-negative women (*n* = 96) who attended cervical cancer screening were included. The prevalence of high-risk HPV was higher in HIV-positive women (*p* = 0.014): high-risk HPV infection in HIV-positive cases was 34 (77%), and in HIV-negative women was 53 (55%). Overall, the prevalence rates of HPV and high-risk HPV were 74% (*n* = 104) and 63% (*n* = 90), respectively. VIA/VILI screening was positive for 20 subjects: 1 had normal histology, 6 had CIN1, 5 had CIN2 or higher, 5 had cervicitis, and no definite diagnosis for 3 women. Among patients with cervical lesions (*n* = 11), 7 were positive for high-risk HPV, and only 1 was HIV-positive. The prevalence of HSV-2 was higher in HIV-positive cases (*n* = 37, 84%) compared to HIV-negative women (*n* = 29, 32%) (*p* < 0.0001). Similarly, HSV-2 was higher in subjects who had high-risk HPV (*n* = 47, 56%) than in women without high-risk HPV infection (*n* = 19, 37%) (*p* = 0.035).

**Table 5:** Recently published clinical studies that documented a correlation between cervical cancer pathogenesis and the impact of local microorganisms with particular reference to the role of *C. trachomatis*.

Study Details	Key Results
George Onyango et al., 2025 [272]. A cross-sectional study in Kenya, which screened coinfections with HIV, HSV-2, and *C. trachomatis* in women with cervical abnormalities (*n* = 517).	The prevalence of CIN was 18.4%. Coinfections with HIV or HSV-2 were two times more likely to test positive for CIN in comparison with uninfected subjects. Similarly, *C. trachomatis* infected women were three times more likely to test positive for CIN.
Klein et al., 2024 [273]. A cross-sectional study from seven centers in Ethiopia was conducted to examine asymptomatic pregnant women (*n* = 779).	*C. trachomatis* and HSV-2 were significantly more common among women who were positive for HPV (any, *n* = 257) and high-risk HPV (*n* = 172).
Loonen et al., 2024 [274]. This study from the Netherlands analyzed the prevalence of pathogenic organisms from 500 high-risk HPV-negative and 492 high-risk HPV-positive cervical smears.	*C. trachomatis*, *M. genitalium*, and bacterial vaginosis * had a significantly higher prevalence in high-risk HPV-positive smears in comparison to high-risk HPV-negative samples.
Wang et al., 2024 [275]. A multicenter cross-sectional study in China, which analyzed HPV-negative (*n* = 261) and HPV-positive (*n* = 270) women with normal or abnormal cervical histology.	As opposed to HPV-negative women, in the HPV-positive group, there were significantly higher incidence rates of lower genital tract infections with *C. trachomatis*, gonococcus, *trichomonas*, as well as mycotic infection.
Disi et al., 2023 [276]. The study examined cervical specimens from 719 women who were referred for colposcopy in China. Among them, 615 were high-risk HPV-positive, and 104 were high-risk HPV-negative.	*U. parvum* and HSV-2 had significantly higher prevalence in high-risk HPV-positive women. Among HPV-16 positive (*n* = 165) and HPV-18 positive (*n* = 54) women, the prevalence of *N. gonorrhoeae* infection was significantly higher compared to other patients. The infection rate of *C. trachomatis* was significantly higher in LSIL or milder cases.
Kaliterna et al., 2023 [277]. This study was conducted in Croatia on 1050 asymptomatic women and found 107 (10.2%) women were positive for high-risk HPV.	A total of 40 women had an abnormal PAP result; 16 of them were positive for high-risk HPV. Statistically significant associations were found between high-risk HPV positivity and infections with *U. urealyticum*, *C. trachomatis*, and *G. vaginalis*.
Ortiz Segarra et al., 2023 [278]. This cross-sectional study collected materials by endocervical brushes from 396 sexually active women from the indigenous communities of Ecuador.	An infection rate of 28.28% was recorded for any type of HPV, 23.48% for high-risk HPV, and 10.35% for low-risk HPV. A statistically significant association was detected between high-risk HPV and *C. trachomatis* infections.
Wang et al., 2023 [279]. This study from China managed genital tract infections of women with high-risk HPV (*n* = 246) and without high-risk HPV (control group, *n* = 354), and analyzed their cervical samples (secretion and exfoliated cells).	Multivariate logistic regression analysis showed that *U. urealyticum* and *C. trachomatis* were independent risk factors for high-risk HPV infection.
Adhikari et al., 2022 [280]. This was a community-randomized trial on HPV-vaccinated women at ages 18.5 and 22 years in Finland. The total number of participants at 18.5 years of age was 11,701, and at 22 years of age were 6618.	At the first visit, 940 had squamous intraepithelial lesions (HPV-16/18 negative: 886). Without lesions, 480 were positive for HPV-16/18. Out of 11,701 participants, the cytological results were missing for 781. On the second visit, 129 had lesions (HPV-16/18 negative: 106, missing: 21). Without lesions, 49 were positive for HPV-16/18, and the cytological results were missing for 1133 (out of 6489 participants). The risk of intraepithelial lesions among HPV vaccinated women was increased significantly with *C. trachomatis* infection and prolonged use of oral contraceptives.
Yu et al., 2022 [281]. This retrospective study included 132 patients from a hospital in China and found 104 were positive for HPV (high-risk type: *n* = 88).	The multivariable analysis showed that infections with HPV-16, *C. trachomatis*, and *M. hominis* were independent risk factors for cervical precancerous lesions.
Mosmann et al., 2021 [282]. This cross-sectional study from Argentina included 50 women with normal cervical cytology and 50 women with abnormal cervical cytology (from a maternity hospital) to assess the status of oral and genital HPV and *C. trachomatis* infection. In the abnormal group, 9 had HSIL, and 41 had LSIL.	HPV DNA was detected in 27% of women (27/100), 18 from genital samples and 14 from oral samples; both mucosal samples were positive in 5 women—3 from the normal group. The most common type was HPV-16 in both normal and abnormal cytology; it was the only type in oral mucosa. HPV was detected more frequently in the normal group (*n* = 18) than in the abnormal group (*n* = 9). Conversely, *C. trachomatis* DNA was detected more frequently in the abnormal group (*n* = 30) than the normal group (*n* = 19). From genital samples, *C. trachomatis* was detected in 35 women, and in 31 from oral samples; both mucosal sites were infected in 17 women—8 from the normal group. Both HPV and *C. trachomatis* significantly correlated with cytological status. However, HPV and *C. trachomatis* coinfection was seen in 14 women—7 had normal cytology.
Xie et al., 2021 [283]. A retrospective study in China, which included 668 patients from a gynecology department—of which 415 women were positive for HPV.	Logistic regression analysis showed that HPV-positive cases were significantly associated with *C. trachomatis*, *U. parvum* (serotypes 3 and 6), and *M. hominis*. Whereas *M. genitalium* and *U. parvum* (aforesaid serotypes) significantly correlated with CIN.
Chen et al., 2020 [284]. This study in China collected cervical swab samples from three groups of women: apparently healthy women who came for a routine checkup (*n* = 1006), women who came for assistance at the reproductive support center (*n* = 666), and women who visited gynecology outpatient clinics (*n* = 3334). The total number of participants was 5006.	Overall, the prevalence of infection with HPV was 15.5% (778/5006), *C. trachomatis* was 4.7% (236/5006), and coinfection was 1.2% (59/5006). For the apparently healthy women (asymptomatic), infection with HPV was 10.8% (109/1006), *C. trachomatis* was 3.8% (38/1006), and coinfection was 0.6% (6/1006). A higher prevalence of infection with *C. trachomatis* was found in HPV-positive women compared to HPV-negative women; a similar phenomenon was also noticed in the opposite way.
Escarcega-Tame et al., 2020 [285]. This cross-sectional study in Mexico was performed on endocervical samples obtained from 189 women with suspected infection. In fact, 184 had an infection with HPV, *C. trachomatis*, or both.	Fifty-six women were positive for HPV (one or more genotypes), 77 were positive for *C. trachomatis*, 51 had both infections, and 5 were not infected. There was an association between HPV and CIN1 (*n* = 22). HPV–*C. trachomatis* coinfection showed a correlation with the development of CIN1 (*n* = 31) and CIN3 (*n* = 7). However, only *C. trachomatis* infection was associated with cervicitis (*n* = 25). In this study, 60 women had CIN1, 11 had CIN3, and 40 women were suffering from cervicitis.

* Bacterial vaginosis: Bacterial vaginosis-associated bacteria include three unclassified bacterial species (BVAB-1, BVAB-2, and BVAB-3). Using sequence homology and phylogenetic analysis, the definite species of BVAB-1 has been found to be *Clostridiales* genomospecies, BVAB-2 aligned with *Oscillospiraceae* bacterium, and BVAB-3 with *Mageeibacillus indolicus*, although other taxa are also present [286]. CIN: Cervical intraepithelial neoplasia, HSIL: High-grade squamous intraepithelial lesion, LSIL: Low-grade squamous intraepithelial lesion, PAP: Papanicolaou smear for cytological test. *C. trachomatis*: *Chlamydia trachomatis*, *G. vaginalis*: *Gardnerella vaginalis*, *M. genitalium*: *Mycoplasma genitalium*, *M. hominis*: *Mycoplasma hominis*, *N. gonorrhoeae*: *Neisseria gonorrhoeae* (*gonococcus*), *U. parvum*: *Ureaplasma parvum*, *U. urealyticum*: *Ureaplasma urealyticum*.

**Table 6:** An overview of prokaryotic 16S ribosomal RNA and equivalent eukaryotic 18S ribosomal RNA genes.

Prokaryotic system	Analysis of the 16S ribosomal RNA (rRNA) gene is an excellent method for the detection and proper identification of any bacteria, apart from the usefulness of this method in the determination of phylogenetic connections among bacteria. In infections, the isolate might be really responsible for the disease or could be present without causing any harm or as a contaminant. Therefore, precise pathogen identification is important for an appropriate treatment strategy. It may be worth mentioning that 16S rRNA gene analysis is a powerful method for the identification of new pathogenic bacteria, non-cultured bacteria, phenotypically aberrant or rarely isolated strains, slow-growing organisms, fastidious pathogens, and bacteria that are poorly described or poorly distinguished by conventional approaches [289,290]. Fortunately, 16S rRNA analysis methods are currently available in many clinical microbiology laboratories due to the growing accessibility of molecular biology techniques such as PCR and sequencing facilities. The bacterial rRNA genes precisely consist of 5S, 16S, and 23S (as well as intergenic regions). With regard to the 16S rRNA gene, which is a part of the ribosome’s small subunit, this gene is usually around 1.5 kb long (i.e., roughly 1500 nucleotides) and has a number of important functions such as protein synthesis, positioning of ribosomal proteins, and supporting the binding of the 50S and 30S subunits [291]. In addition, all bacteria contain at least one copy of the 16S gene, which encompasses different sequences that include highly conserved, variable, and hypervariable regions. For each bacterial species, the hypervariable regions are unique and thus used for bacterial classification. On the other hand, the conserved regions are utilized to develop universal primers, which can bind to known sequences (common in other bacteria) [292]. By and large, in our routine laboratory practice, these universal primers are designed to target the first ~500 base pairs of the 16S rRNA gene, since the analysis of this portion (V1–V3 regions) is usually thought satisfactory for precise detection of most bacterial species [291]. Nevertheless, the 16S rRNA gene can be a target for a number of antibacterial drugs, and mutations in this gene may influence bacterial susceptibility to these drugs; thus, phenotypic resistance to these drugs can be distinguished by the 16S rRNA gene sequencing [293,294,295]. Therefore, analysis of the 16S rRNA gene perhaps has a direct impact on therapeutic management.
Eukaryotic system	Like the utilization of 16S rRNA gene characteristics in the prokaryotic system, 18S rRNA in eukaryotic cells is a common molecular marker. Different studies reported that analysis of 18S rRNA is very useful in the detection of various protozoal infections/presence—for example, *Trichomonas vaginalis* and a range of vaginal fungal species such as *Candida albicans*, *Candida glabrata*, and *Saccharomyces cerevisiae* [296,297,298]. Like 16S rRNA of the small subunit, 18S rRNA (1.8 kb) is a part of the small 40S ribosomal subunit, while 5S, 5.8S, and 28S rRNAs constitute the large 60S subunit (complete ribosome with ribosomal proteins—80S in eukaryotes compared to 70S in prokaryotes). Both conserved and variable regions are present in the 18S rRNA gene. Sequencing of the 18S rRNA gene, particularly with the internal transcribed spacers (ITS: intervening noncoding regions), is advantageous in molecular diagnosis [299].

**Table 7:** Selected recent studies that have demonstrated an adverse effect on *Lactobacillus* in the environment of vaginal dysbiosis and other pathological conditions such as cervical dysplasia and cancer.

Investigators	Study Design	Salient Observations
Guo et al., 2025 [309]. China	Between June 2021 and June 2022, vaginal DNA samples were collected from 151 Uygur and Han women—Han controls (*n* = 22), HPV transient infection (*n* = 26), and persistent infection group (*n* = 28), as well as Uygur controls (*n* = 28), transient infection (*n* = 17), and persistent infection group (*n* = 30).	Ethnic-specific differences in vaginal microbes were observed. In Han women, *Sneathia* increased significantly in persistent HPV cases. In Uygur women, *Gardnerella*, *Streptococcus*, *Prevotella*, and *Shuttleworthia* increased significantly in the transient infection group, with lower *Lactobacillus* in comparison with other groups.
Li and Wu 2025 [310] China	DNA was extracted from vaginal samples of normal controls (*n* = 16), HPV-positive women (*n* = 16), CIN1 (*n* = 14), CIN2 (*n* = 6), CIN3 (*n* = 15), and cervical cancer patients (*n* = 2) between December 2018 and September 2020.	*Lactobacillus* was the prevalent bacterium in all groups, and the prevalence rates are: 70.9% in normal controls, 60.2% in HPV-positive women, 63.9% in CIN1, 97.7% in CIN2, 52.0% in CIN3, and 36.9% in cervical cancer. An increased proportion of *Gardnerella* was detected with HPV infection, possibly associated with the progression of cervical lesions.
Parvez et al., 2025 [311] India	Between December 2018 and April 2022, vaginal swabs were collected, and DNA extraction was performed in 58 subjects—62% were HPV-positive and 38% were HPV-negative. Among the HPV-positive group, symptomatic cases were 57%, and 39% were asymptomatic cases.	*Lactobacillus* showed a high abundance in HPV-negative samples (76.7%), although lower in HPV-positive samples (60.3%). *Gardnerella* was significantly higher in HPV-positive (22.4%) than HPV-negative (10.0%). Similarly, *Coriobacteriaceae*, *Prevotella*, *Aerococcus*, and *Clostridium* had elevated levels in HPV-positive samples. Interestingly, *L. iners* was more prevalent in HPV-positive, while *L. helveticus* was dominant in HPV-negative samples.
Asensio-Puig et al., 2024 [312] Spain	Out of 22 pairs of cervicovaginal samples (liquid-based cytology samples and self-collected specimens), 14 samples met the sequencing quality criteria, and 8 were HPV-positive.	Compared to HPV-negative samples, HPV-positive samples documented a lower abundance of *Lactobacillus*, but a higher abundance of *L. iners*, and a high bacterial diversity that specifically included *Atopobium*, *Parvimonas*, *Mageeibacillus*, *Sneathia*, *Megasphaera*, *Peptoniphilus*, and *Dialister*. In the HPV-negative cases, a higher abundance of *L. crispatus* and *L. gasseri* were detected.
Fan et al., 2024 [313] China	From June to December 2020, enrolment of 125 subjects who underwent HPV test, cytology test, and colposcopy. There were 27 normal women, 40 with high-risk HPV infection, 40 with CIN and 18 cervical cancer patients.	The diversity of vaginal bacterial species in cervical cancer patients was primarily associated with a decrease in *Lactobacillus* and *Cyanobacteria* and was also correlated with an enhanced number of *Dialister* and *Peptoniphilus*. Compared to normal controls and the high-risk HPV group, the relative abundance of *Cyanobacteria* in the CIN group was significantly decreased.
Jimenez et al., 2024 [314] United States	The study included 100 primarily Hispanic and non-Hispanic White premenopausal women. There were 20 HPV-negative and 31 HPV-positive women without dysplasia, 12 with low-grade lesions (LSIL), 27 with high-grade lesions (HSIL), and 10 with invasive cervical cancer (samples were not available from one participant).	*Atopobiacaeae* colonization was associated with increased vaginal pH, vaginal dysbiosis, and decreased *Lactobacillus* dominance. A higher prevalence of *Atopobiaceae* was detected among Hispanic subjects as well as women with higher gravidity and parity. In cervical cancer, *Atopobiaceae* and particularly *Fannyhessea vaginae* had a higher prevalence. *Atopobiacaeae* were positively correlated with vaginal microbiome, such as *Anaerococcus*, *Dialister*, *Prevotella*, *Sneathia*, and *Bifidobacterium*/*Gardnerella* *, as well as with pro-inflammatory cytokines such as IL-1α, IL-1β, and TNFα. (* *Gardnerella vaginalis* belongs to the *Bifidobacteriaceae* family)
Łaniewski et al., 2024 [315] United States	This pilot study recruited 31 women (16 Native American and 15 non-Native women) between December 2020 and April 2022.	Women with vaginal dysbiosis had higher vaginal pH and were more frequently infected with high-risk HPV. There was also an association between high-risk HPV and *Gardnerella vaginalis* infections. Women with higher levels of *Lactobacillus* had normal vaginal pH and tended to have HPV-negative status. *L. crispatus* (not *L. iners*) was negatively associated with bacterial vaginosis-associated bacteria, e.g., *Gardnerella*, *Fannyhessea*, *Prevotella*, *Sneathia*, and *Megasphaera*. Interestingly, vaginal dysbiosis was linked with larger household size, lower education level, and high parity.
Liu et al., 2024 [316] China	Cases were collected from the Gynecology and Obstetrics department between Mar 2022 and Mar 2023. HPV-positive cases were 241, and HPV-negative cases were 1759.	A significant deficiency in *Lactobacilli* was noticed in HPV-positive cases compared to the HPV-negative group. Moreover, in comparison to HPV-negative subjects, the results showed that bacterial vaginitis and aerobic vaginitis were closely associated with HPV-positive cases.
Ou et al., 2024 [317] China	Based on the histological assessment (from January to October 2021), the subjects were categorized as follows: normal controls (without neoplasia, *n* = 10), CIN1 (*n* = 15), CIN2 (*n* = 25), CIN3 (*n* = 25), and invasive cancer (*n* = 25).	Among controls, *L. crispatus* was dominant. Compared to controls, the proportion of *Lactobacillus* was reduced with the progression of cervical dysplasia. In cancer patients, the abundance of *L. johnsonii* and *L. iners* was higher, and taxa such as *Atopobiaceae*, *Prevotellaceae*, and *Streptococcaceae* were dominant.
Yang et al., 2024 [318] China	The study subjects were married women who visited the gynecology outpatient clinic between September 2019 and February 2020. Participants were categorized into five groups: persistent high-risk HPV without cervical pathology (*n* = 20), with low-grade lesions (LSIL, *n* = 20), high-grade lesions (HSIL, *n* = 20), squamous cancer (*n* = 20), and HPV-negative controls (*n* = 19).	In the non-infected controls, the predominant bacteria were *Lactobacillus*, *Gardnerella*, *Prevotella*, and *Streptococcus*. On the other hand, the vaginal bacterial composition in subjects of all groups with HPV showed alterations that were predominantly associated with a reduction in *Lactobacillus* and an increase in *Gardnerella* when compared with the normal HPV-negative control group. The findings revealed a decrease in *Lactobacillus* and an increase in *Gardenerella* subsequent to persistent HPV infection.
Yu et al., 2024 [319] China	Subjects diagnosed with cervical lesions for the first time were included. There were 30 healthy controls, 29 with low-grade squamous intraepithelial lesions, 33 with high-grade lesions (HSIL), and 29 cervical cancer patients.	Compared to the other groups, decreased *Lactobacillus* abundance was documented in cancer patients. The abundance of *Acinetobacter*, *Bacillus*, *Corynebacterium*, *Escherichia*, *Fenollaria*, *Peptoniphilus*, *Staphylococcus*, and *Streptococcus* showed a rising trend with the degrees of cervical lesions and cancer.
